# Emotional reactivity and decision-making under social deprivation: the role of trait anxiety during quarantine

**DOI:** 10.1098/rsos.251773

**Published:** 2026-10-14

**Authors:** Avisha, Akhilesh Tayade, Arjun Ramakrishnan

**Affiliations:** ^1^ Department of Psychology, University of Southern California, Los Angeles, CA, USA; ^2^ Department of Biological Sciences and Bioengineering, Indian Institute of Technology Kanpur, Kanpur, Uttar Pradesh, India

**Keywords:** EMA, COVID-19, affect, decision-making, quarantine, generalized linear mixed models, social interactions

## Abstract

Social isolation measures enforced during the COVID-19 pandemic highlighted the importance of the social environment in shaping affective states and decision-making. However, the day-to-day experience and psychological recovery from social deprivation, especially among individuals with high trait anxiety, remains poorly understood. We conducted a 10-day longitudinal study with 116 university students returning to campus, collecting approximately 31 000 momentary assessments of affect and social interactions during a 3-day quarantine and subsequent period. Quarantine increased the need for social interaction and altered patterns of social engagement, whereas trait anxiety was robustly associated with reduced positive affect and elevated negative affect. Close interactions following quarantine were associated with higher positive affect. We also administered a virtual patch-foraging task to participants during and after quarantine. Greater variability in negative affect was associated with lower patch exit thresholds, suggesting a tendency to persist in depleted patches. These findings highlight how trait anxiety shapes emotional experience and social behaviour in daily life and reveal how fluctuations in negative affect can influence sequential decision-making under uncertainty.

## 1. Introduction

Humans are inherently social beings who thrive in environments rich in connections and interactions [1–4]. Such interactions profoundly shape mood [5,6], decision-making [7–9] and mental and physical health [10–14]. When social interactions are disrupted, the resulting isolation and loneliness can have wide-ranging psychological consequences, increasing risk for anxiety, depression and suicidal behaviour [15–19]. Because affective states also influence how individuals evaluate rewards, risks and opportunities [20–24], disruptions to social environments may have downstream consequences not only for emotional well-being but also for decision-making.

These vulnerabilities became particularly evident during the COVID-19 pandemic. Quarantine and distancing measures drastically curtailed social engagement, increased loneliness and disrupted daily routines, contributing to widespread anxiety and other mental health problems [25–34]. While studies during this period highlighted the importance of social interaction for mental health, less is known about the moment-to-moment psychological impact of social deprivation, the process of emotional recovery once restrictions are lifted and whether such affective changes influence decision-making in dynamic environments.

To address these gaps, we conducted a 10-day study of young adults quarantined on a university campus after travel, with the first 3 days spent in isolation upon returning to campus, followed by 7 days in their university dorms. During these 10 days, we used ecological momentary assessment (EMA) [35] to capture fine-grained fluctuations in affect and social context, with participants reporting their affect, interactions and daily events four times per day. We hypothesized that reduced interactions during quarantine would contribute to decreased positive affect and increased negative affect, and that participants would spend more time interacting with close others after quarantine, with affective rebound following renewed social engagement.

Because psychological response to social context varies widely across individuals, we focused on trait anxiety—a stable disposition linked to negative affectivity and heightened risk for mood and anxiety disorders [36–40]. Individuals high in trait anxiety show greater emotional reactivity and stronger dependence on social context [33,41,42]. Yet, few naturalistic studies have examined how it shapes emotional responses to periods of social isolation and subsequent reintegration. We, therefore, hypothesized that individuals with higher trait anxiety would report greater negative affect and reduced positive affect during both quarantine and post-quarantine periods, and that they may also derive greater emotional benefit from increased social interaction following quarantine [33,34,42–45].

Finally, because affect influences decision-making [20–24], we examined its behavioural consequences using a virtual patch-foraging game. Foraging provides a naturalistic framework for explore–exploit trade-offs [46,47], where agents must decide whether to persist with a depleting resource or seek alternatives. Prior work suggests that positive affect promotes exploration, whereas negative affect biases decisions towards cautious exploitation [48–50]. Participants completed the task once during quarantine and once afterwards, enabling us to test whether momentary affect and trait anxiety predicted decision patterns in this ecologically grounded paradigm. We hypothesized that increased stress and negative affect during quarantine would reduce patch residence times during foraging. Together, this study leveraged the natural experiment of quarantine to investigate how social deprivation and its resolution influence affect, how trait anxiety modulates these processes and how transient emotional states spill over into real-world decision-making.

## 2. Methods

This study was conducted in 2021 during the period of declining COVID-19 cases following the second wave in India. A total of 116 participants completed the study (79 identified as male, 36 as female and 1 preferred not to disclose gender). Participants were independently recruited via email from among students returning to the on-campus residence of IIT Kanpur, across seven batches (B1–B7) over four months (see figure 1a).

**Figure 1.**
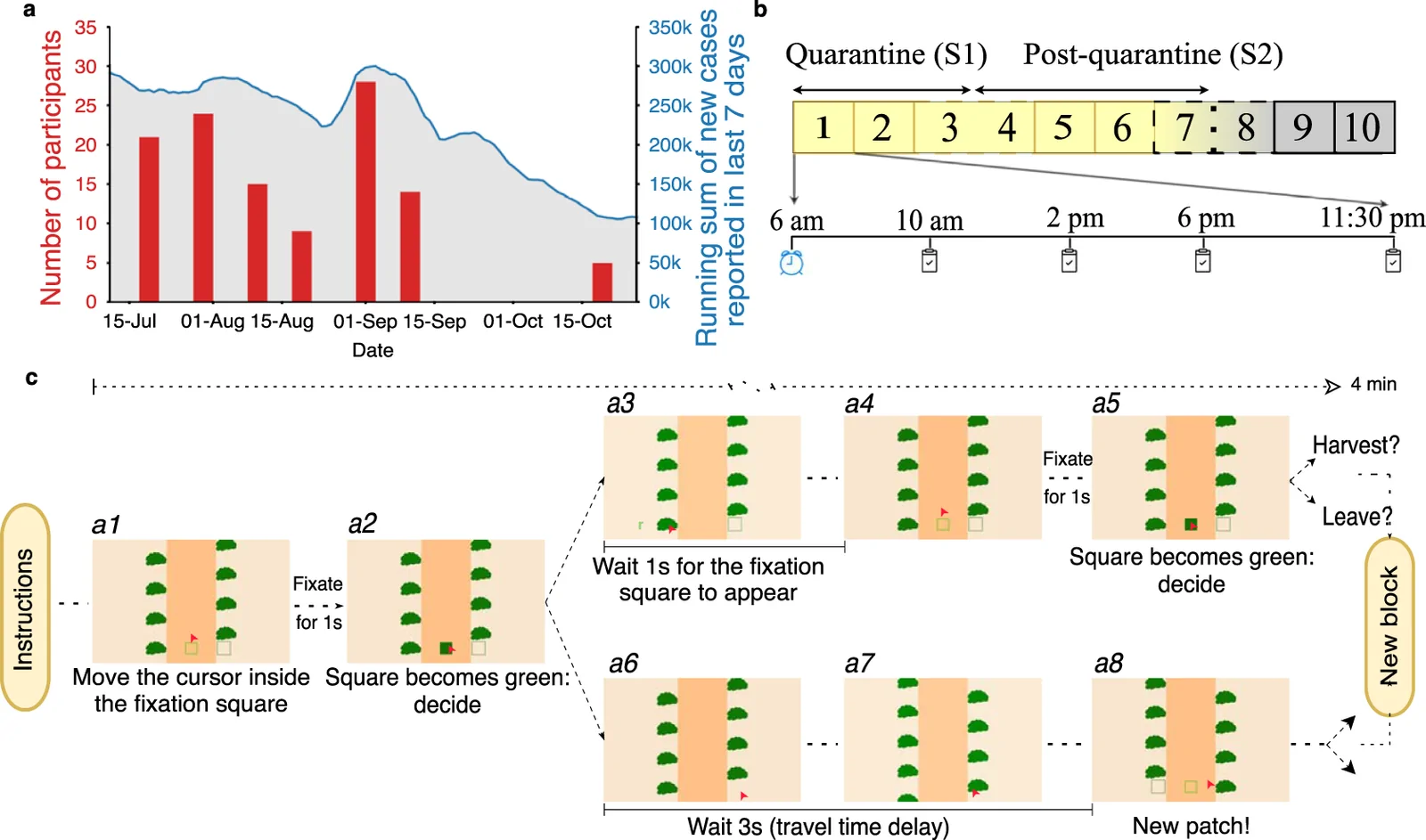
Study design. (a) Number of participants in different batches. Participants were IIT Kanpur students who returned to the institute campus from across the country in batches on different dates and underwent quarantine upon their arrival. The ordinate shown in blue displays the running sum of COVID-19 cases over the moving window of 7 days in the country (data source: WHO COVID-19 dashboard at https://data.who.int/dashboards/covid19/data). (b) Deterministic schedule of EMA surveys sent via the mobile app. S2: 4 days after quarantine (S1) ended. (c) Schematic of the computer-based patch-foraging task. The task consisted of two blocks (‘short’ and ‘long’). Each block lasted for 4 min. On each trial, participants decided to harvest a patch (a3) or leave it (a6) for the next one: Participants received uncertain rewards on each harvest, determined by a reward function unknown to them. If participants chose to leave a patch, an animation played that mimicked travelling to a new patch (a6–a8). Participants did not receive any reward if they chose to leave a patch in a given trial. The inter-patch travel time remained fixed in a block but changed between blocks: the travel time was 3 s in the short block and 10 s in the long block. Actual cursor appearance depended on the participant's computer configuration.

Participants completed a baseline survey administered online through Qualtrics prior to participation. The baseline survey included the Trait Anxiety subscale (Form Y-2) of the State-Trait Anxiety Inventory (STAI) for Adults [36] along with additional questions (see supplementary material, §1.1).

### 2.1. Ecological momentary assessments

#### 2.1.1. Data collection

We collected ecological momentary data using the Ethica app (Ethica Data Services, Inc.), a proprietary platform that delivered automated in-app surveys on mobile phones. The app provided a dashboard displaying pending surveys and sent push notifications to prompt timely responses.

Each day, participants were prompted to complete four routine surveys and one sleep-related survey, totalling five surveys per day (see figure 1b for prompt timings). Thus, participants received 50 surveys over 10 days. Routine surveys remained active for 90 min after the initial notification. If a routine survey was not completed within the first hour, a reminder notification was sent. The daily sleep survey (see supplementary material, §1.3) was delivered at 06.00 and remained open for 24 h to answer.

The routine survey, designed to be completed in approximately 3–4 min, assessed participants’ social interactions, affect, desire for social contact, naps and experiences of stressful and positive events. Social interactions were assessed by asking participants who they were interacting with at the time of the survey (‘alone’, ‘someone not as close to me’ or ‘someone close to me’), which we refer to as *Interaction-with*. We asked participants whether the reported interaction was face-to-face or digital (text-based, video or phone call). The desire for social interaction (‘need to be with others’) was assessed on a five-point Likert scale with responses consisting of *not at all*, *maybe*, *slightly*, *definitely* and *most definitely*. Momentary affect was assessed using items indexing positive affect (‘How happy/joyful/cheerful are you feeling at the moment?’) and negative affect (‘How anxious/worried/nervous are you feeling at the moment?’), each answered on a five-point Likert scale containing options *not at all*, *slightly*, *moderately*, *very* and *extremely*. Questions asked in routine EMA surveys and corresponding response options are provided in detail in table S1 in the supplementary material.

Before the study, participants were provided a training video introducing the study procedures, survey schedule and instructions for using the Ethica app. They were advised to enable push notifications to ensure prompt survey responses and were instructed to avoid completing surveys under the influence of alcohol.

#### 2.1.2. Preregistration and deviation

We preregistered our study procedures on AsPredicted: https://aspredicted.org/3WF_WSP. We aimed to test seven hypotheses in this study (H1–H7). However, sleep-related analyses (H3) were excluded from the current study because they would require substantial additional analyses and would considerably broaden the scope of the paper.

In addition, we did not pursue H5, which aimed to examine gender-based differences, owing to an imbalanced gender distribution in our sample (approx. 33% female participants). This imbalance would have resulted in limited statistical power and unstable estimates for gender-based comparisons. Following [51], we have provided a detailed disclosure of deviations from preregistration in table S18 in the supplementary material.

#### 2.1.3. Study timeline and segmentation

Upon arrival on the campus, participants underwent institutional quarantine for at least 3 days (see figure 1b). Those who tested negative for COVID-19 at the end of this period were permitted to move into their residential hostels. We designate the participants’ arrival day on campus as ‘Day 0’ for the purpose of the study. For example, if a participant returned on 20 August 2021, their EMA surveys commenced on the morning of 21 August 2021.

We used the responses from quarantine surveys to estimate when participants moved out from quarantine to their residence hostels (see supplementary material, §1.2 and tables S2–S5 for details): we refer to the data containing only participants who reported their quarantine exit day as ‘Exit_R_’ and the data containing participants for whom we estimated quarantine exit day as ‘Exit_R/E_’.

We refer to the days participants spent in quarantine as ‘S1’, the first 4 days after S1 as ‘S2’ and the remaining days of the study after S2 as ‘S3’. Given our primary interest in the immediate effects of quarantine, the analyses in this study based on EMA responses focused on understanding the differences between S1 (quarantine) and S2 (post-quarantine).

#### 2.1.4. Statistical analysis of ecological momentary assessment data

We used a set of exclusion criteria before analysing the data (see figure 2 and supplementary material, §2.1). Handling grouped ordinal data with linear mixed-effects models [52] or models that assume a Gaussian distribution for the response variables can lead to biased parameter estimates [53,54]. Therefore, we modelled routine EMA survey responses with cumulative link mixed models (CLMMs) implemented via the R package *ordinal* [55]. We first fitted CLMMs (with logit link function and unstructured thresholds) to model changes in participants’ need to interact with others (‘Interaction-intensity’) and identity of social interaction partners (‘Interaction-with’) as a function of trait anxiety and social context. We used models with specifications as follows:

(2.1)Response ∼ TA + SC + (1 | Participant),(2.2)Response ∼ TA × SC + (1 | Participant),

where ‘TA’ represents the min–max normalized trait anxiety score (continuous variable), and ‘SC’ denotes the quarantine versus post-quarantine setting (categorical variable).

**Figure 2.**
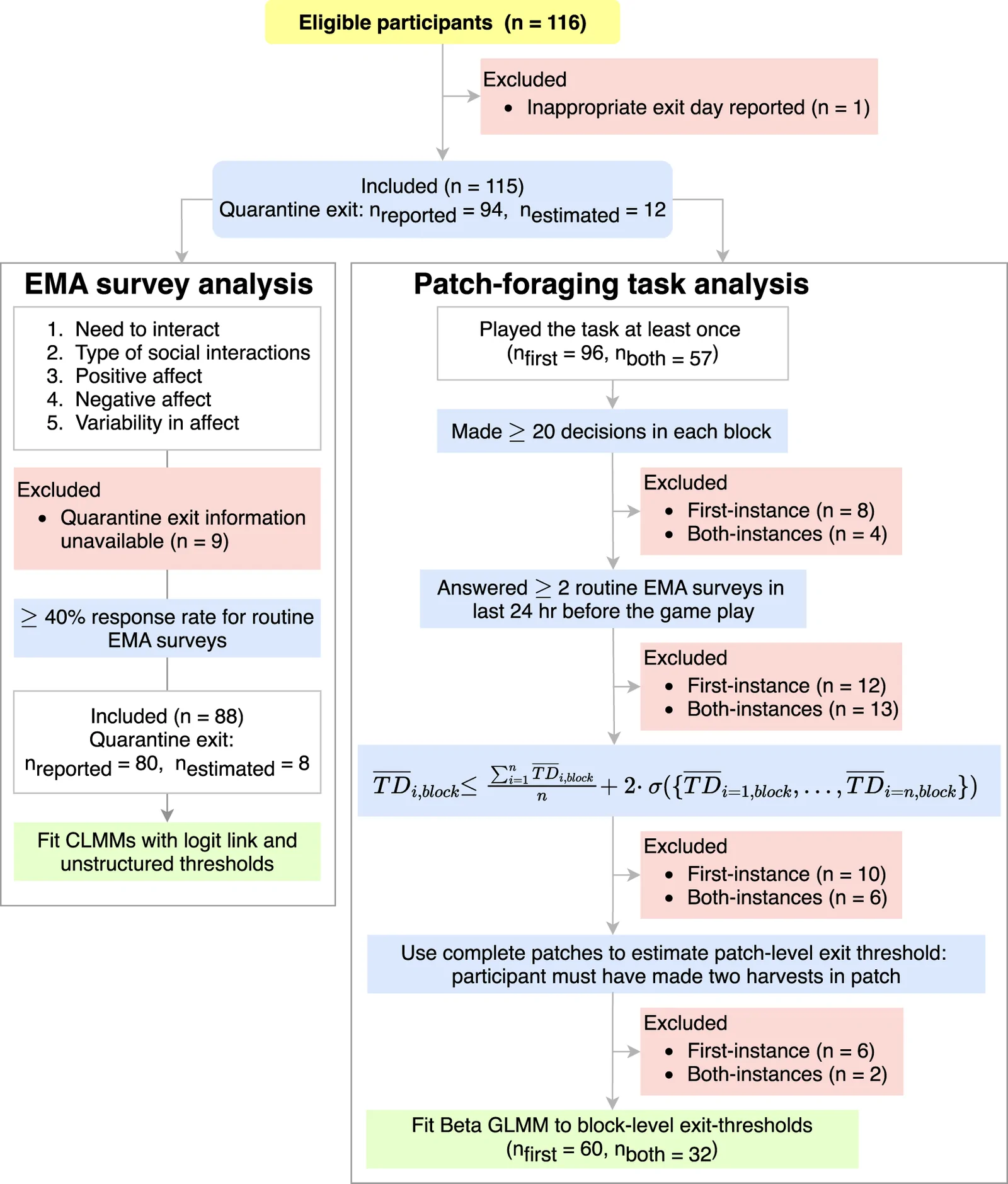
Exclusion criteria used in data preprocessing. The study period of 10 days was considered to calculate response rate for routine EMA surveys; *n*_first_ and *n*_both_ represent the number of participants who played the task at least once during the study period and during both quarantine and post-quarantine setting, respectively. CLMM: cumulative link mixed model, GLMM: generalized linear mixed model, *TD*: trial duration (in seconds), ${\overset{―}{TD}}_{i,\;block}$: average trial duration in a (short or long) block for *i*th participant and ***σ***: standard deviation.

In addition, we also fitted the CLMMs to both Interaction-intensity and Interaction-with where the effect of social context on response could vary across participants:

(2.3) $$\begin{array}{r} Response\;∼\;TA\;+\;SC\;+\;(1\;+\;SC\;|\;Participant). \end{array}$$

To investigate how social interactions, along with trait anxiety and social context, influenced affective experience, we fitted CLMMs to model positive and negative affect ratings using the following specifications:

(2.4)Response ∼ InteractionWith × TA × SC + (1 | Participant),(2.5)Response ∼ InteractionWith × TA × SC + (1 + SC | Participant).

We fitted CLMMs (equations (2.1)–(2.5)) to both Exit_R/E_ and Exit_R_ and observed that model estimates were not sensitive to the consideration of imputation procedure (see supplementary material, §3.6).

#### 2.1.5. Variability in affect

We used the coefficient of unalikeability [56] as a metric for variability in positive and negative affect:

(2.6) $$\begin{array}{r} XA_{unalike}=\frac{\sum_{i\neq j}^{q}c\left(x_{i},x_{j}\right)}{q^{2}\;-\;q}, \end{array}$$

with $c(x_{i},\;x_{j})\;=\;\{\begin{array}{cl} & 0\;if\;x_{i}=\;x_{j}\\ & 1\;if\;x_{i}\neq \;x_{j} \end{array}$ where *XA* is either positive affect or negative affect, *x_i_* is the encoded EMA survey response for the affect variable and *q* is the number of routine EMA surveys the participant answered within the given social context. The coefficient captured how often survey responses changed from one to another, focusing on the qualitative aspect of ordered responses. Such formulation of variability seems more sensible compared to standard deviation and mean-squared successive difference, which assess ‘how much’ measurements vary from each other, as the formulation of the coefficient does not rely on computing averages from ordered responses and also does not assume that differences between any two adjacent categories are equal [54].

As the coefficients of unalikeability take values between 0 and 1 (both ends inclusive), we fitted ordered beta generalized linear mixed models (GLMMs) to them, calculated for quarantine and post-quarantine periods using the Exit_R/E_ responses of positive and negative affect (for model specifications, see equations (2.1) and (2.2) in §2). In addition, we refitted these models to the coefficients using the Exit_R_ responses of positive and negative affect. Models with random slopes for social context did not converge to stable estimates; therefore, we excluded them from the analysis. The simpler model (equation (2.1) in §2) had better fits to both Exit_R/E_ and Exit_R_ compared to the model containing the interaction term for trait anxiety and social context (equation (2.2) in §2) (see supplementary material, table S17).

### 2.2. Foraging game

#### 2.2.1. Data collection

We administered participants a version of the patch-foraging task (figure 1c) in [51] once during quarantine and once during a post-quarantine setting (see supplementary material, §3.1 for a detailed task description). Briefly, the task began with instructions, followed by a short demonstration video showing how participants were supposed to make harvest or leave decisions. Following the video, the display changed to show a series of patches, indicating entry into a foraging block: patches were close together in the short block and farther apart in the long block. We administered participants with either a ‘shortLong’ arrangement or a ‘longShort’ arrangement: each block lasted for 4 mins. Participants harvested patches serially: each harvest earned a reward, displayed next to the patch. The first harvest in a patch yielded a reward *r*_0_, with

(2.7) $$\begin{array}{r} r_{0}=7\;+N\left(\mu \;=\;0,\sigma \;=\;0.25\right) \end{array}$$

and each subsequent harvest decreased the reward according to

(2.8) $$\begin{array}{r} r_{i}\;=r_{i-1}-0.5\;+N\left(0,\;\sigma \;=\;0.25\right), \end{array}$$

where *N*(µ, σ) was a realization of Gaussian distribution with expected value µ and standard deviation σ. Once depleted, patches yielded no further rewards (i.e. *r_i_* ≥ 0 for *i* ≥ 0).

We excluded some data based on a set of criteria, resulting in a sample of 66 participants (see figure 2 for an overview, supplementary material, §3.3 and figs. S7 and S8 for details). Before applying the trial-duration-based exclusion criterion, we corrected measurement error in the first trial durations (see supplementary material, §3.2).

#### 2.2.2. Statistical analyses of the first-game instance

We analysed block-level average-estimated exit thresholds for the reward of patches that a participant could leave before the 4-min time limit (block duration) was reached; we refer to such patches as complete patches. For each participant, we calculated the block-level exit threshold (*u*_exit_) by averaging over patch-level exit thresholds. For each complete patch, we estimated the patch-level exit threshold to be the average of the last two rewards in the patch. Of the 66 participants considered, six participants harvested each patch at most once in one of the two blocks they had played (see supplementary material, fig. S9). Thus, we could not estimate block-level thresholds for these participants for either block, which led to their removal from further consideration, resulting in the data with *n = 60*. We transformed block-level thresholds to ${\bar{u}}_{exit}\;\in (0,\;1)$ because of the theoretical properties of the reward function used in the task (see supplementary material, §3.4 and fig. S10 for details).

The beta GLMM, with random intercepts for each participant, was fitted to ${\bar{u}}_{exit}$ using the R package *glmmTMB* [57]. The formula of beta GLMM was specified as follows:

(2.9) $$\begin{array}{r} {\bar{u}}_{exit}\;∼\;TravelTime+EnvOrder+TA\;+PA_{median}\\ +\;NA_{median}+PA_{unalike}+NA_{unalike}+\left(1|\;Participant\right), \end{array}$$

where TravelTime was the inter-patch travel time in the corresponding block (encoded as a categorical variable: ‘0’ = 3 s, ‘1’ = 10 s), EnvOrder was the arrangement of blocks (longShort or shortLong, encoded as a categorical variable), TA was the min–max normalized trait anxiety score obtained from STAI (Form Y-2), and XA_median_ and XA_unalike_ were the median response and coefficient of unalikeability, respectively, computed from routine EMA surveys completed by the participant in the 24 hours before gameplay (XA indicates positive affect or negative affect; PA: positive affect, NA: negative affect). We opted for the 24-h pre-game window to maximize sample size while simultaneously capturing the task-day-like affect (but see supplementary material, table S18, Unregistered Step #1).

We performed model diagnostics on the beta GLMM (see supplementary material, §3.5 and fig. S11). We observed that the estimated fixed-effects coefficients were similar to those from the model refitted without considering participants with extreme decision-making (cf. supplementary material, table S10 with table S12). When we retained these participants in the data and computed confidence intervals using standard robust errors from the refitted beta GLMM, we obtained similar evidence for associations between regressors and the exit threshold (see supplementary material, §3.7 and fig. S13, green segments). Here, beta GLMM allowed us to discuss participant-level changes in the exit thresholds as a function of regressors (see supplementary material, fig. S12). However, we preferred to use marginal effects obtained from beta GLMM using *GLMMadaptive* [58] for our discussion, as they represent population-averaged effects (see supplementary material, table S11).

## 3. Results

We conducted this study during the COVID-19 pandemic with college students who returned to campus following an extended lockdown. Upon arrival, participants were quarantined for 3–4 days as a precautionary measure to reduce the risk of SARS-CoV-2 transmission. Before moving out of quarantine, students underwent a COVID-19 test. Those who tested negative were allowed to exit quarantine and return to their residence hostels. After applying exclusion criteria, we analysed data from 88 young adults who reported their mood, social interactions and experiences of pleasant/unpleasant events over a 10-day period, including days spent in quarantine (see figure 2).

### 3.1. Quarantine, trait anxiety and the need for social interaction

To explore whether quarantine affected participants’ desire to be around others, we assessed their need for social interaction during each momentary assessment using the item ‘*Do you want to be with other people right now?*’ rated on a five-point Likert scale (see figure 3a). Higher scores on the scale indicated a stronger desire for social interaction. In addition, participants completed the STAI-Y2, which measured trait anxiety. We hypothesized that the quarantine period, which involved limited social interactions, would increase the need for social interaction, and that this effect would be more pronounced for individuals with higher trait anxiety.

**Figure 3.**
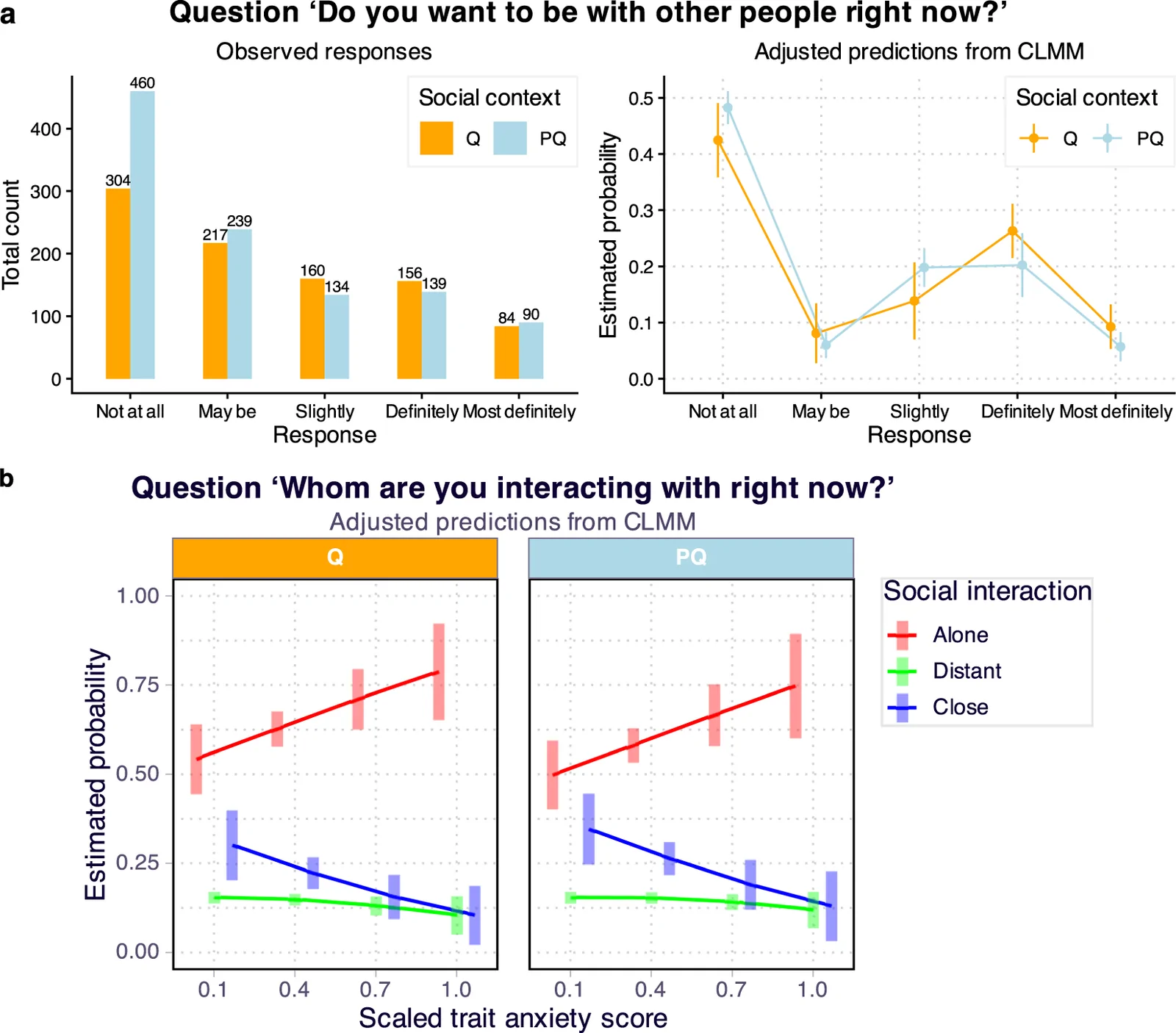
Observed responses from EMA surveys and predictions from the cumulative link mixed models (CLMMs). Q: Quarantine, PQ: post-quarantine. (a) Histogram denotes the counts of different responses to the question asked in routine EMA surveys. Data are aggregated across all participants (*n* = 88). Bias-corrected adjusted predictions are shown from the CLMM with social context (Q versus PQ) and scaled trait anxiety score (continuous variable) as predictors and participants as random effects (see §2, equation (2.1)). Only the conditional model (i.e. estimates of fixed-effect coefficients) was used to obtain linear predictions, with the scaled trait anxiety score held constant at 0.435. Bias-correction was then applied using the standard deviation of random effects (= 1.976) to obtain cumulative probabilities of each response category, and subsequently, response probabilities were obtained. (b) Bias-corrected adjusted predictions obtained using only the conditional model from the fitted CLMM to the responses of ‘InteractionWith’ (see §2, equation (2.1)) are shown. We chose four values of trait anxiety scores to understand how increase in trait anxiety was associated with changes in social interactions across different social settings (quarantine versus post-quarantine). Error bars are 95% Wald confidence intervals around the estimated probabilities.

To test this hypothesis, we employed a CLMM with random intercepts only (see §2, equation (2.1)) with ‘need to be with others’ as the target variable, and trait anxiety score and social context (quarantine versus post-quarantine) as predictors (see supplementary material, §2.2).

The results revealed that participants were more likely to report a greater need to be with others during the quarantine period compared to the post-quarantine period (log-odds estimate: −0.526, 95% CI: [−0.705, −0.348], *p* < 0.001; see supplementary material, table S6 and figure 3a). Moreover, individuals with higher trait anxiety were more likely to express a stronger need for social interaction than those with lower trait anxiety, although the association was less evident statistically (log-odds estimate: 2.430, 95% CI: [−0.042, 4.902], *p* = 0.054; see supplementary material, table S6).

To evaluate the robustness of our findings, we fitted a CLMM where each participant could have a different slope for social context, with logit link function and unstructured thresholds (§2, equation (2.3)). The model with random slopes for social context provided evidence for the associations between trait anxiety and social anxiety that were qualitatively similar in nature to the evidence provided by the random-intercept-only model (§2, equation (2.1)) (see supplementary material, table S6), despite providing better fits to the responses of need to be with others generated from Exit_R/E_ and Exit_R_ (see supplementary material, table S17)_._

These findings suggest that quarantine increased individuals’ need for social interaction, when compared to the post-quarantine setting, indicating that the social context created by quarantine might have been insufficient to satisfy participants’ social needs. Furthermore, trait anxiety was found to be weakly associated with this heightened need for interaction, with individuals experiencing higher anxiety being more likely to report a stronger desire to be with others in both contexts.

### 3.2. Quarantine, trait anxiety and the type of social interactions

To further investigate how the need for social interaction manifests, we examined the time participants spent alone versus with others during both the quarantine (Q) and post-quarantine (PQ) periods. Specifically, we categorized social interactions based on responses to ‘*Who are you interacting with right now?*’, distinguishing between being alone, interacting with ‘close’ others or interacting with ‘distant’ others.

We hypothesized that, following quarantine, participants would spend more time with people they felt close to than with distant individuals (see H1).

Upon analysing the data, we found that the total number of reports (supplementary material, fig. S3; 922 reports in Q and 1062 in PQ) for interactions with close partners did not differ much between the quarantine and post-quarantine contexts (supplementary material, fig. S3; close interactions: Q – 620; PQ – 619). However, the cumulative number of reports of interactions with close partners during quarantine was higher than the cumulative number of reports of interactions with distant others during the same period (see supplementary material, fig. S3; interactions: close – 221; distant – 81). This suggests that, in a socially restrictive context, individuals tend to interact more with close others compared to distant others. Interestingly, the most notable shift in social interactions occurred in the transition from quarantine to post-quarantine, particularly in the time spent interacting with distant others (see supplementary material, fig. S3; distant interactions: Q – 81; PQ – 220).

Participants reported spending significantly more time alone in both quarantine and post-quarantine settings (see figure 3b and supplementary material, S3; Q: 620 out of 922 instances which is 67.2%; PQ: 619 out of 1062 which is 58.3%).

To statistically validate changes in social interaction and to explore the role of trait anxiety in shaping these interactions, we modelled the impact of trait anxiety and social context (quarantine versus post-quarantine) on the type of social interaction reported (alone versus interacting with close or distant others). Using a CLMM with random intercepts (see equations (2.1) and (2.2) in §2, and supplementary material, §2.2), we found that participants with higher trait anxiety were more likely to report being alone rather than interacting with others (log-odds estimate: −1.602, 95% CI: [−3.107, −0.098], *p* = 0.037) (see supplementary material, table S7 for model estimates; see also figure 3b). This result suggests that individuals with higher trait anxiety were more inclined to withdraw and spend time alone compared to their low trait-anxiety counterparts.

Furthermore, we observed that participants were more likely to report interacting with others (close and distant individuals) in the post-quarantine setting compared to the quarantine setting (log-odds estimate: 0.258, 95% CI: [0.061, 0.455], *p* = 0.010) (see figure 3b; see supplementary material, table S7 for model estimates). This finding underscores the shift in social dynamics following the easing of quarantine measures, with greater opportunities for social engagement outside of quarantine.

When examining the nature of social interactions, we found that high trait-anxious individuals were not only more likely to be alone but they were also less likely to interact with distant others compared to their close partners (see figure 3b; notice how the blue curve is always higher than the green counterpart even at scaled trait anxiety score of 0.7: with regard to quarantine setting, for scaled trait anxiety score of 0.1, the estimated probabilities are 0.155 [95% CI: 0.139, 0.171] and 0.302 [95% CI: 0.204, 0.400] for distant and close interactions, respectively, while at 0.7 score, the probabilities are 0.132 [0.105, 0.159] and 0.157 [0.095, 0.219]). This result indicates that higher levels of trait anxiety were likely to influence the type of social interactions, with anxious individuals potentially opting for more emotionally supportive interactions with people they were close to.

Among the models (equations (2.1)–(2.3) in §2) fitted to the responses of type of social interactions, we observed that the model with random slope (equation (2.3)) was the best, followed by the random-intercept-only model with additive terms for TA and SC (equation 2.1), for both Exit_R/E_ and Exit_R_, according to likelihood ratio tests (see supplementary material, table S17). Despite this, the evidence for the associations between trait anxiety and social context with the type of social interactions was qualitatively similar (see supplementary material, table S7).

In summary, while participants were more likely to report being alone than interacting with others in both contexts, the transition from quarantine to post-quarantine was associated with a higher likelihood of social interaction. In addition, individuals with higher trait anxiety were more likely to be alone but likely to be with close partners over distant others when engaged in a social interaction. These results highlight the interplay between trait anxiety and social interaction, particularly in the context of changing social environments.

### 3.3. Changes in affective states across contexts and their relationship to trait anxiety

Next, we explored the momentary affective states reported by participants during both the quarantine and post-quarantine periods. Positive affect was measured with the item ‘*How happy/joyful/cheerful are you feeling at the moment?*’ and negative affect with the item ‘*How anxious/worried/nervous are you feeling at the moment?*’, each rated on a five-point scale from 0 (not at all) to 4 (extremely). Specifically, we aimed to investigate how participants’ emotional experiences changed across these two distinct social contexts and how their trait anxiety influenced these affective states.

We hypothesized that participants would experience higher levels of negative affect and lower levels of positive affect during quarantine (see H2). In addition, we predicted that individuals with high trait anxiety would experience higher levels of negative affect and lower levels of positive affect compared to those with lower trait anxiety, regardless of the social context (see H4).

Both positive and negative affect ratings spanned the full response range, though responses were more frequent at lower intensity levels (see supplementary material, figs. S4 and S5). We analysed these data using CLMMs with random intercepts, including a three-way interaction between trait anxiety, social context and interaction type (see §2, equation 2.4).

Contrary to our hypothesis (H2), negative affect was not associated with transition from quarantine to post-quarantine setting (negative affect: log odds = −0.530, 95% CI: [−1.259, 0.199], p = 0.154), while positive affect was weakly associated with change in social context (positive affect: log odds = −0.542, 95% CI: [−1.143, 0.058], *p* = 0.077) (see supplementary material, fig. S6 and tables S8 and S9).

**Figure 4.**
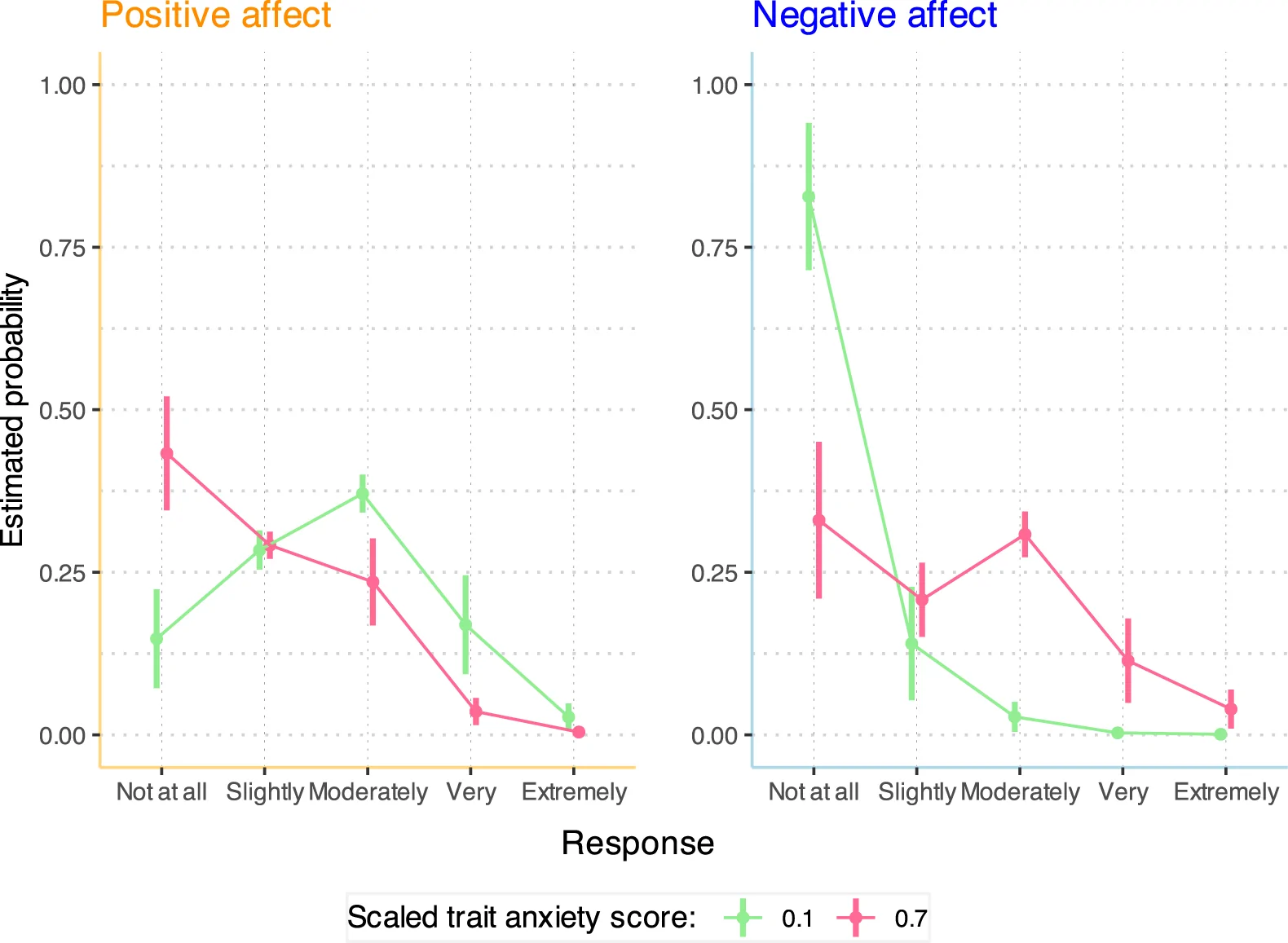
Adjusted predictions using fitted cumulative link mixed models (CLMMs) with random intercepts for survey responses of positive and negative affect across quarantine and post-quarantine settings. Model estimates are reported in tables S8 and S9 in the supplementary material. Low trait-anxious individuals (green) displayed higher positive affect and lower negative affect. The predictions were obtained from CLMMs with the term ‘SocialContext’ taking values of 0 (= quarantine) and 1 (= post-quarantine) and trait anxiety, TA, fixed at 0.1 and 0.7; the predictions were averaged over the levels of Interaction-with. Only the conditional models (i.e. estimates of fixed-effect coefficients) were used to obtain linear predictions, which were then bias-corrected to obtain cumulative probabilities, and subsequently, probabilities of each response category. Error bars are 95% Wald confidence intervals around the estimated probabilities.

Consistent with our hypothesis (H4), higher trait anxiety was robustly associated with increased negative affect (log odds = 5.860, 95% CI: [3.520, 8.199], *p* < 0.001) and decreased positive affect (log odds = −3.642, 95% CI: [−5.520, −1.764], *p* < 0.001) across both contexts (see figure 4 and supplementary material, fig. S6). These findings indicate that individuals with elevated trait anxiety experienced more negative and less positive affect persistently, irrespective of social context.

Finally, given that participants were more likely to engage in social interactions in the post-quarantine context, we also sought to investigate whether these interactions influenced affective states as a function of trait anxiety. We hypothesized that individuals with high trait anxiety would experience greater improvements in emotional well-being—such as increased positive affect and decreased negative affect—owing to the resumption of social interactions after quarantine (see H6). Specifically, we expected that participants with high trait anxiety would report more positive affect and less negative affect when interacting with close or distant partners post-quarantine, compared to the quarantine period.

Close interactions during post-quarantine were associated with higher positive affect compared to similar interactions during quarantine (log odds = 1.429, 95% CI: [0.191, 2.668], *p* = 0.024; Social Context [PQ] × Interact with [Close] in the supplementary material, fig. S6 and table S8). However, there was no change in negative affect when close interactions across these social contexts were compared (log odds = 0.850, 95% CI: [−0.713, 2.412], *p* = 0.287; also see supplementary material, table S9 and fig. S6).

Contrary to our hypothesis (H6), the positive affect of high trait-anxious individuals did not change based on the interactions with close others (log-odds estimate: −0.979, 95% CI = [−4.693, 0.735], *p* = 0.153; Scaled TA × Social Context [PQ] × Interact with [Close] in the supplementary material, fig. S6 and table S8). Similarly, the negative affect of high trait-anxious individuals did not change based on close interactions going from quarantine to post-quarantine period (log-odds estimate = −2.060, 95% CI = [−5.310, 1.191], *p* = 0.214, see Scaled TA × Social Context [PQ] × Interact with [Close] in the supplementary material, fig. S6 and table S9).

To assess the robustness of our findings, we fitted CLMMs with random slopes for social context to positive and negative affect (see §2, equation (2.5)). For both Exit_R/E_ and Exit_R_, the random slopes model provided a better fit than the random-intercept-only model (equation (2.4)) for positive as well as negative affect, as per likelihood ratio tests (see supplementary material, table S17). The primary associations between trait anxiety, social context and affect remained stable across specifications (see supplementary material, tables S8 and S9 and figs. S15 and S16 for visual comparison). Following quarantine, close interactions were associated with increased positive affect; however, this association (i.e. Social Context [PQ] × Interact with [Close]) was less stable across different models (see supplementary material, tables S8 and S9 and figs. S15 and S16). These results suggest that while trait anxiety is robustly associated with affective states, context-dependent effects may be more sensitive to model specification.

We then explored the relationship between trait anxiety and social context with variability in positive and negative affect (see §2, variability in affect). As per the model fitted to Exit_R/E_ (see §2, variability in affect and equation (2.1)), variability in positive affect was only weakly associated with social context (log-odds estimate: −0.175, 95% CI: [−0.357, 0.007], *p* = 0.060) and did not change with trait anxiety (log-odds estimate: 0.027, 95% CI: [−0.733, 0.788], *p* = 0.943). While variability in negative affect did not change across social contexts (log odds: −0.030, 95% CI = [−0.249, 0.189], *p* = 0.789), it was significantly associated with trait anxiety (log odds: 1.599, 95% CI = [0.789, 2.409], *p* < 0.001). When we used the data Exit_R_ instead of Exit_R/E_, we observed similar results when we refitted these random-intercept-only models to the coefficients of unalikeability (see supplementary material, table S19).

In summary, trait anxiety was associated with positive and negative affect in different ways. In contrast, the effect of social context on affect was weaker than initially hypothesized. We also observed that, while the evidence for an association between affect variability and social context was weak, high trait anxiety was strongly associated with increased variability in negative affect but was not associated with variability in positive affect. Together, these findings paint a nuanced picture in which momentary affect is more reliably associated with trait anxiety rather than short-term changes in social context.

### 3.4. Affect and decision-making in the patch-foraging task

So far, we have explored how self-reported affect changes as a function of social context, trait anxiety and social interactions. We further examined the influence of momentary affective states on decision-making in a virtual patch-foraging task (see foraging game in §2). The task consisted of virtual patches that participants visited serially by making a ‘stay’ (harvest) decision or a ‘leave’ decision on each trial to maximize reward collection, as each harvest decision resulted in a non-negative reward (see equations (2.7) and (2.8) in §2). The task consisted of a short block and a long block; the block type determined how long the participant had to wait after leaving a patch before reaching the next one. The inter-patch travel time was 3 s in the short block and 10 s in the long block. Previous research has shown that affective experiences, such as stress, can lead to suboptimal foraging strategies, such as overharvesting patches relative to an optimal policy [48]. In this study, we asked participants to complete the patch-foraging task twice: once during quarantine and once after exiting quarantine (see figure 5a).

**Figure 5.**
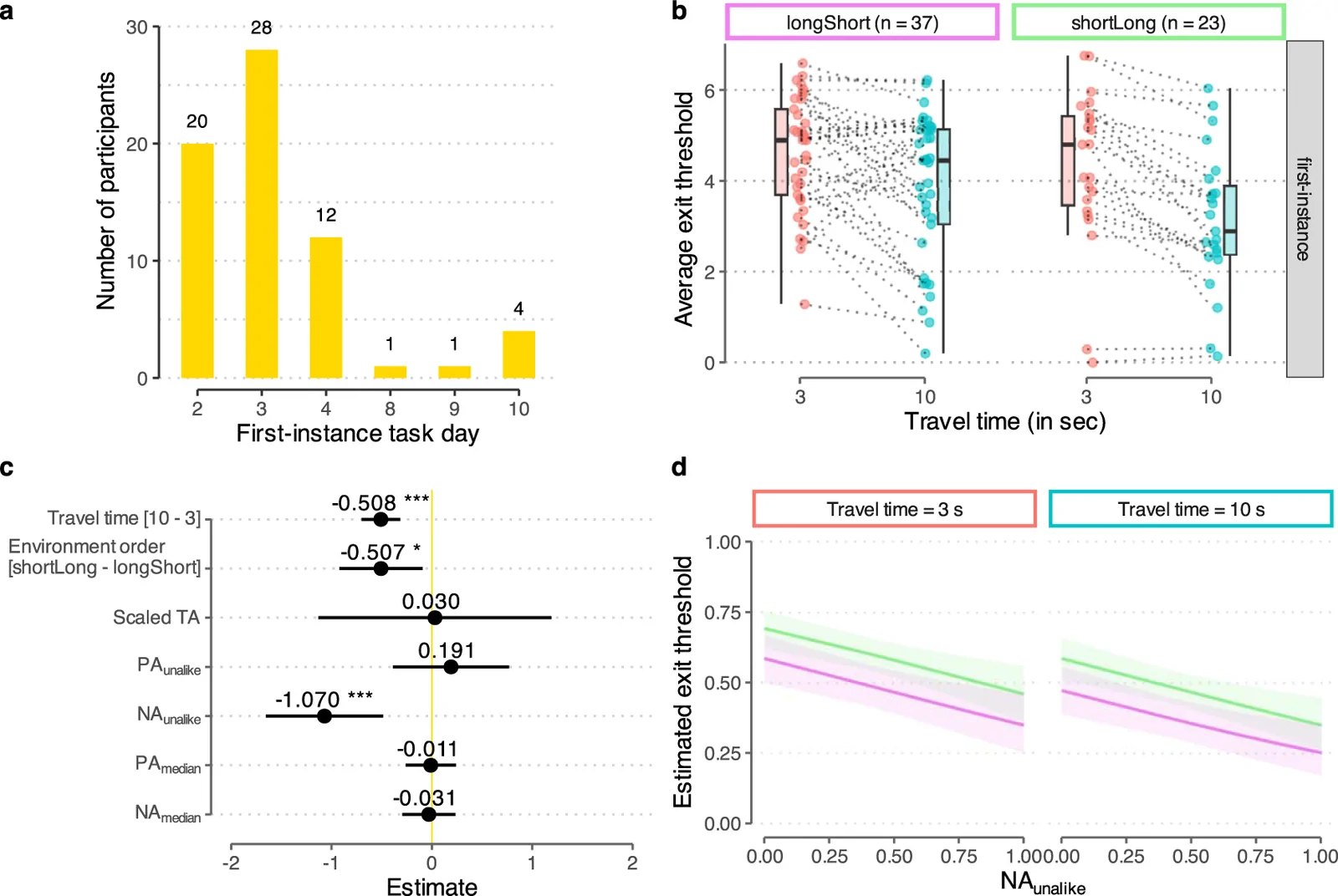
Patch-foraging task inference. (a) Participants played the patch-foraging task for the first time on different days during the study duration of 10 days (*n* = 66). (b) Distribution of block-level exit thresholds versus travel time in the block (*n* = 60): thresholds could not be estimated for six participants (see supplementary material, §§3.3,3.4). Participants played one of the two arrangements: longShort (pink) or shortLong (green). Half-violin plots and box plots are plotted using the sample displayed next to it as coloured circles; some circles have been moved horizontally to prevent overlap. Points connected with a dotted line belong to the same participant. (c) Estimates of fixed-effect coefficients (except the fixed-intercept) from the beta GLMM fitted to the data of ${\bar{u}}_{exit}$ from the first-game instance of the patch-foraging task (*n* = 60). Error bars are 95% Wald confidence intervals around the estimates (see [59] for details on estimation procedure) * *p* < 0.5, *** *p <* 0.001. (d) Bias-corrected adjusted predictions from fitted beta GLMM with random intercepts fitted to the data from the first-game instance of the patch-foraging task. Predictions were obtained over a grid containing combinations of different values of NA_unalike_ {0, 0.4, 0.5, 0.67, 0.83, 1}, environment order (green: longShort and pink: shortLong) and travel time (3 s or 10 s) while keeping other predictor variables from the model at fixed values: PA_unalike_ = 0.983, PA_median_ = 0.983, NA_median_ = 0.667 (see supplementary material, table S15). Only the conditional model of the beta GLMM was used to obtain linear predictions, which were bias-corrected to obtain predictions of block-level exit thresholds. The coloured lines connect the estimated probabilities, and the coloured regions connect the lower and upper 95% Wald confidence intervals around them.

We began by analysing data from participants’ first-game instance (see supplementary material, §§3.3‒3.5). Similar to the methodology of Lenow *et al.* [48], we calculated patch exit threshold. This threshold marks the point at which the participant determines the patch is no longer worth foraging and moves to a new patch. We also approximated the average exit threshold for each participant as a proxy for the perceived environmental richness of a given block (see foraging game in §2). Specifically, participants with a high average exit threshold left patches early, indicating they perceived the environment to be of higher quality, whereas participants with a low average exit threshold left patches later, perceiving the environment as less rewarding.

The analysed dataset consisted of 120 observations: two block-level average exit thresholds, one for each of the 60 participants (see figure 5b). To assess whether participants adapted their foraging strategies in response to experimental manipulations of inter-patch travel time, we first applied the marginal value theorem, which posits that foragers should stay in patches longer when the travel time between patches is higher. We modelled changes in block-level exit thresholds as a function of inter-patch travel time using a beta GLMM (see foraging game in §2, and figure 5c, supplementary material, tables S10 and S11 for estimated coefficients). As expected, we observed a decrease in the exit threshold with an increase in inter-patch travel time (marginal beta GLMM, see supplementary material, table S11; log odds: −0.466, 95% CI: [−0.607, −0.326], *p* < 0.001), demonstrating that participants adjusted their foraging strategies in line with the marginal value theorem.

We also counterbalanced the order in which participants experienced the different inter-patch travel time conditions (see ‘Block order’ in supplementary material, §3.1). Some participants played the longer travel time block first (longShort arrangement), while others played the shorter travel time block first (shortLong arrangement). Previous research has shown that participants often perform asymmetrically when transitioning from a low-resource to a high-resource environment, as opposed to the reverse order [60]. In line with these findings, we observed that, on average, block-level thresholds were lower for the shortLong arrangement, indicating greater sensitivity to changes in the environment (marginal beta GLMM; log odds: −0.466, 95% CI: [−0.906, −0.025], *p* = 0.038).

We also examined how trait anxiety was associated with foraging behaviour. A previous study has suggested that higher trait anxiety is associated with reduced time spent in patches [61]. However, in this analysis, the effect of trait anxiety on block-level exit thresholds was not significant (marginal beta GLMM; log odds: 0.040, 95% CI: [−0.996, 1.075], *p* = 0.940).

Having validated the participants’ game performance with respect to travel time, block order and trait anxiety, we then sought to understand how momentary affective states influenced decision-making in the patch-foraging task. We tested this hypothesis by comparing two models: one that included affect-related regressors (see equation (2.9) in §2) and one that did not. The model with affect-related regressors fit the data better, indicating that momentary affective states were associated with participants’ patch-foraging decisions (likelihood ratio test with beta GLMMs; logLikelihood_affect_ = 50.169, logLikelihood_no-affect_ = 43.019, $\chi_{\left(4\right)}^{2}$ = 0.006). When we examined the impact of positive affect, we found that both positive affect and its variability were not significantly associated with block-level exit thresholds (both *p* > 0.5, see supplementary material, table S11). However, negative affect exhibited a more complex relationship with decision-making. While the overall level of negative affect did not influence the exit thresholds (marginal beta GLMM; log-odds estimate: −0.026, 95% CI: [−0.273, 0.221], *p* = 0.839), the variability in negative affect had a significant effect. Specifically, when participants experienced higher variability in negative affect before playing the task, their block-level exit thresholds were lower (marginal beta GLMM; log odds: −0.981, 95% CI: [−1.497, −0.466], *p* < 0.001). This result suggests that participants who experienced higher variability in negative affect were more likely to leave patches later, even when the reward potential was clearly on the lower side, indicating a tendency to harvest smaller rewards (see figure 5d).

The associations between travel time, environment order and negative affect variability with exit thresholds were consistent when we repeated the analysis without participants we deemed highly influential on the beta GLMM fit because of their low random-intercept values (see supplementary material, figs. S11 and table S12) for both Exit_R/E_ and Exit_R_ (see supplementary material, §3.6 and fig. S13).

To assess robustness of results to data exclusion criteria, we conducted additional analyses retaining all participants and using robust standard errors, as well as relaxing key inclusion criteria (e.g. minimum trial counts and EMA aggregation windows) while simultaneously considering the effect of the imputation procedure for the quarantine exit day on the results (see supplementary material, §3.7). The effect of travel time on exit thresholds remained consistent across all specifications (see supplementary material, fig. S17). While we observed a significant association between negative affect variability and exit thresholds in the 24-h pre-game window, no such association was observed in the 12-h window. However, we again observed the effect when we computed variability using the 36-h pre-game window and task-day responses with at least 20 trials per block (supplementary material, figs. S17‒S20). Note that larger windows (24 and 36 h) yielded higher sample sizes than shorter windows (task-day and 12 h) and thus led to tests that were more powerful at detecting the effect if it existed (see supplementary material, fig. S20).

Although our preregistration specified analyses based on patch stay duration, distributional properties of these data (strictly positive and right-skewed) violated assumptions of linear (Gaussian) mixed-effects models (see supplementary material, §3.8 and figs. S21‒S23). We, therefore, analysed the derived exit-threshold measure [48], which provides a bounded, theoretically grounded index of foraging decisions (see supplementary material, fig. S10 and §3.4) and, as discussed above, is well summarized by the beta GLMM. Importantly, modelling block-level average patch stay duration with a gamma GLMM yielded qualitatively similar results, indicating that the key effects are robust to the choice of behavioural metric (see supplementary material, §3.8 and figs. S24‒S27). This convergence suggests that, for the study's primary exclusion criteria (figure 2), our findings reflect underlying decision processes rather than metric-specific ones.

To further characterize the decision process underlying prolonged patch residence, we fitted a drift-diffusion model [62] jointly to stay–leave choices and response times (see supplementary material, §3.9 and table S16). Drift rate increased with immediate patch reward (*B* = 1.56, s.d. = 0.03, 95% CI: [1.50, 1.61]) and with longer travel times (*B* = 0.02, s.d. = 0.007, 95% CI: [0.004, 0.03]), consistent with stronger evidence favouring continued exploitation when local rewards were high or alternatives were more costly. Importantly, greater variability in negative affect was also associated with higher drift rates favouring staying (B = 0.54, s.d. = 0.22, 95% CI: [0.12, 1.07]), mirroring the results of the exit-threshold analyses. These findings suggest that prolonged patch residence was associated with altered value-evidence accumulation rather than with simple motor perseveration or fatigue alone.

We also repeated the foraging task analysis with data from a subset of participants who played the task in both contexts (quarantine and post-quarantine). After applying the exclusion criteria (figure 2), the final sample included 32 participants (see supplementary material, §3.10). As in the initial analysis, we found that higher variability in negative affect was associated with lower block-level exit thresholds (marginal beta GLMM; log odds: −0.399, 95% CI: [−0.776, −0.023], *p* = 0.038) (see supplementary material, tables S13 and S14).

In summary, we found that higher variability in negative affect experienced in the time period before playing the task was associated with lower block-level exit thresholds. This reduction in exit threshold was in the same direction as the one observed for increased travel time. These findings suggest that volatile negative affect might lead individuals to perceive the foraging environment as poorer in quality, which, in turn, results in longer average patch stays. Note, however, that the true patch quality is unchanged, unlike when the travel time changes. These findings provide insights into how emotional variability, especially in negative affect, can significantly influence decision-making in dynamic, resource-based tasks such as patch foraging.

## 4. Discussion

This study provides new insights into how short-term social deprivation during COVID-19 quarantine, followed by resumption of normalcy, impacted affective dynamics and decision-making in a real-world context.

As expected, individuals with high trait anxiety experienced elevated stress, increased negative affect and reduced positive affect, extending previous findings on the adverse emotional consequences of trait anxiety [41]. Interestingly, transitioning from quarantine to post-quarantine was weakly associated with positive affect, but no association of social context with negative affect was observed. By integrating a virtual patch-foraging task with EMA, we further demonstrated that experience of negative affect states of unequal intensity before the task was associated with prolonged patch residence and lower exit thresholds during the task, indicating a tendency towards delayed exploration and persistent exploitation, linking affective variability to real-world decision-making under uncertainty.

### 4.1. Trait anxiety, social engagement and emotional functioning in daily life

Our findings provide ecologically valid evidence for how trait anxiety shapes emotional experience and social behaviour in everyday life. Individuals with higher trait anxiety reported diminished momentary well-being, characterized by elevated negative affect and reduced positive affect, consistent with prior work [63–66]. Although work-related activities contributed substantially to pleasant experiences following quarantine (supplementary material, fig. S1), work-related stress also emerged as one of the most reported sources of distress across social contexts (supplementary material, fig. S2).

Trait anxiety was also associated with patterns of social engagement. Highly trait-anxious individuals spent more time alone during both quarantine and post-quarantine periods, potentially reflecting avoidance or social withdrawal tendencies. At the same time, interactions with close others following quarantine tended to increase positive affect, reinforcing previous findings on the protective effects of social closeness and interpersonal support [33,41,42]. Together, these findings suggest that trait anxiety exerts a persistent influence on emotional experience, stress perception and social behaviour in naturalistic settings, while also highlighting the potential buffering role of meaningful social connections.

### 4.2. Social isolation and affective instability during quarantine

Our findings further demonstrate that even a brief period of mandated social isolation can influence emotional functioning. During quarantine, participants reported greater need for social interaction, which declined once restrictions were lifted. Loneliness has been strongly linked to elevated negative affect and emotional dysregulation [15,59,67,68] and may contribute to heightened emotional reactivity, particularly in the absence of meaningful social contact [61,69–71]. These effects have been observed across age groups, cultural settings and both experimental and naturalistic EMA studies. Our results partially support this account. While positive affect fluctuations tended to be more frequent during quarantine and were not associated with trait anxiety, fluctuations in negative affect were strongly associated with trait anxiety. These results suggest that, with a higher likelihood of social interactions following the end of quarantine, affective fluctuations tended to be less frequent during post-quarantine periods, leading to partial emotional re-stabilization, yet remained more frequent among high trait-anxious individuals. Collectively, these findings reinforce the importance of social connectedness for emotional regulation and suggest that even short periods of social deprivation might alter affective dynamics in daily life.

### 4.3. Affective influence on decision-making

To assess how affective states influence behaviour, we used a patch-foraging task as an ecologically valid read-out of explore–exploit decision-making [72,73]. According to the marginal value theorem, individuals should leave a patch when its reward rate falls below the environmental average. While such models often describe normative behaviour, substantial variability has been documented in both human and animal studies [46,74]. Our results contribute to a growing body of work suggesting that affective states can systematically bias these decisions. Acute stress, for instance, has been shown to promote overharvesting by blunting sensitivity to environmental rewards [48], and experimental manipulations of arousal and valence have been shown to shift stay–leave decisions [49,75].

In our study, negative affect variability, but not its average value, was associated with suboptimal foraging behaviour: participants with greater negative affect variability were more likely to overharvest, a finding that was supported by results from the decision process model as well. Interestingly, trait anxiety was not associated with patch exit thresholds in the present dataset. This discrepancy may reflect distinct influences of stable anxiety traits and transient affective states on decision-making. Consistent with this interpretation, trait anxiety was associated with greater variability in negative affect, whereas only affective variability predicted foraging behaviour. Strikingly, the behavioural association of affective variability resembled the effects of increased travel time between patches—both led to longer patch stays and reduced reward-rate sensitivity, possibly owing to disrupted internal estimates of environmental reward. This pattern is consistent with previous findings linking negative affect to diminished cognitive flexibility [49] and narrowed attentional scope [76]. That said, we interpret these findings with appropriate caution. The present experiment was not designed to dissociate altered value sensitivity from other sources of persistence, such as decision inertia or strategic uncertainty. Overall, these results indicate that fluctuations in affect—not sustained negative mood—can reshape reward sensitivity and decision strategies during sequential choice. Although the neural systems governing explore–exploit trade-offs may be shared across paradigms [46,47], their behavioural manifestations probably depend on task structure. More broadly, these results highlight the potential of sequential decision-making tasks as objective behavioural markers of emotional health over time [77].

### 4.4. Strengths and limitations

A major strength of this study is its use of EMA across 10 days, allowing fine-grained analysis of affective dynamics and social behaviour in real time. This longitudinal design avoids the limitations of retrospective recall and offers greater ecological validity than traditional cross-sectional methods. The incorporation of a behavioural task—the patch-foraging game—adds a unique dimension by linking emotional experience to observable decision-making patterns. The four-month data-collection window provided a large, diverse temporal sample, capturing fluctuations during different phases of the post-lockdown transition.

To assess the robustness of our findings, we evaluated alternative exclusion criteria and different quarantine exit-day handling procedures for data generation, and multiple model specifications, particularly random slope models, and used a robust estimation procedure in the presence of potential outliers for EMA variables. Models with random slopes for social context had better fits to data when they converged to stable estimates with positive definite variance–covariance matrices. However, the overall pattern of findings—particularly the associations between trait anxiety and social context and an EMA variable—remained qualitatively similar across specifications. Given the consistency of results across these approaches, we retained the simple, random-intercept-only models for the primary analyses and for visualization of predicted effects, while reporting additional analyses in the supplementary material. Similarly, the task-related associations were robust to alternative exclusion criteria, the presence of influential participants and the choice of behaviour metric (exit threshold versus patch stay duration).

Despite these strengths, we note several limitations of the study. While we focused on affective variability, we did not include a validated measure of emotional dysregulation, which would have allowed for a more direct examination of regulatory mechanisms. Our sample was also limited to students aged 22–26 residing in university dorms, which limits the generalizability of our findings to broader age groups and socio-cultural contexts. Although many psychosocial stressors encountered in this population may mirror those experienced in larger-scale lockdowns (e.g. entire towns or cities), key differences—such as socio-economic status, family responsibilities and community support —must be considered when extending these results to other settings.

In addition, owing to logistical constraints, the study could not adopt a randomized control design: all students underwent mandatory quarantine for the first 3 days. Moreover, we lacked baseline EMA data from pre-quarantine life, limiting our ability to draw strong causal inferences. The emotional variability observed during quarantine was probably influenced not only by isolation but also by pandemic-specific concerns such as health anxiety, academic uncertainty and disrupted routines. Furthermore, only participants who completed at least 40% of the routine surveys were included in the analysis. While this was necessary for data quality, it may have introduced self-selection bias, as individuals who were more emotionally stable or motivated might have been more likely to comply with the study protocol.

In sum, this study highlights the complex interplay between social isolation, trait anxiety, emotional variability and decision-making in young adults navigating quarantine during the COVID-19 pandemic. Our findings provide compelling evidence that momentary emotional fluctuations—not static affect levels—can meaningfully alter real-world behaviour, particularly under conditions of uncertainty. These results underscore the importance of considering both dispositional and contextual factors in understanding mental health and point to the potential of behavioural tasks as tools for monitoring emotional well-being in ecologically valid settings. Future studies can expand these findings to more diverse populations and settings, incorporate causal designs and explore interventions that target affective stability and social connection as pathways to resilience.

## Supplementary Material



## Data Availability

The anonymized data collected via EMA surveys and the patch-foraging task, along with the associated analysis script, are publicly available in an OSF repository at https://osf.io/djm2w [78]. The files that render the patch-foraging task in a web browser and Python code that fits hierarchical drift-diffusion models to the task data can be obtained by contacting the corresponding author. Supplementary material is available online [79].
