# Supplementary material for: Emotional reactivity and decision-making under social deprivation: the role of trait anxiety during quarantine
Source: R Soc Open Sci. 2026 Oct 14;13(10):251773. doi: 10.1098/rsos.251773 (PMC13673250; doi:10.1098/rsos.251773)
Supplement: Supplementary file 1 [file rsos.251773.zip › rsos.251773/rsos251773_si_001.docx]

**Supplementary Information for**

**Emotional Reactivity and Decision-Making Under Social Deprivation: The Role of Trait Anxiety During Quarantine**

Avisha Avisha, Akhilesh Tayade, Arjun Ramakrishnan^*^

^*^Corresponding author. Email: arjunr@iitk.ac.in

# **Table of Contents**

Figure S1	4

Figure S2	5

Figure S3	6

Figure S4	7

Figure S5	7

Figure S6	8

Figure S7	9

Figure S8	10

Figure S9	11

Figure S10	12

Figure S11	12

Figure S12	13

Figure S13	14

Figure S14	15

Figure S15	16

Figure S16.	16

Figure S17	17

Figure S18	18

Figure S19	18

Figure S20	19

Figure S21	20

Figure S22	20

Figure S23	21

Figure S24	22

Figure S25	22

Figure S26	23

Figure S27	23

Table S1	24

Table S2	26

Table S3	27

Table S4	28

Table S5	29

Table S6	30

Table S7	32

Table S8	33

Table S9	35

Table S10.	37

Table S11	37

Table S12	38

Table S13	38

Table S14	38

Table S15.	39

Table S16	40

Table S17	41

Table S18	43

Table S19	49

Section 1: Supplementary notes	50

S1.1 Baseline survey	50

S1.2 Quarantine exit	50

S1.3 Sleep survey.	50

S1.4 Study compensation	50

Section 2: EMA data	51

S2.1 Exclusion criteria for EMA data	51

S2.2 Statistical analysis of EMA data	51

Section 3: Foraging task	51

Section 3.1 Paradigm.	51

Section 3.2 Error correction in the measurement of the first trial duration.	52

Section 3.3 Data exclusion for the first-game-instance of the patch-foraging task	53

Section 3.4 Data transformation	53

Section 3.5 Model diagnostics.	54

Section 3.6 Reanalyses without quarantine exit uncertainty	54

Section 3.7 Sensitivity of foraging results to exclusion criteria and sample attrition	55

Section 3.8 Analysis of patch stay duration	56

Section 3.9 Modeling of reaction time	56

Section 3.10 Analysis of both-game-instances of the patch-foraging task.	57

Supplementary references	57

# **Figure S1**: Histograms displaying the frequency of pleasant events of different nature in quarantine (Q) and post-quarantine (PQ) settings. Data is aggregated across participants from all batches (*n = 88)*. We asked participants to report, using a forced-choice “Yes/No” question, if they experienced any pleasant event before answering the survey. If participants reported having experienced a pleasant event, they could further decide to report the nature of the event by selecting more than one option from the eight-options list. (Top) The number of surveys in which participants did not report experience of pleasant events were higher than those in which they reported occurrence of pleasant events. (Bottom) Zooming on data which represents only the presence of pleasant events helps to highlight the different nature of such events. If the response in the survey assigned multiple attributes to a pleasant event (e.g., ‘financial event’ and ‘work-related event’), we considered both attributes as independent responses contributing to the count of occurrence of pleasure events. However, the number of surveys where participants assigned multiple attributes to pleasant event reported were low (59 out of 497 surveys).

# **Figure S2**: Histograms displaying the occurrence of stressful events of different nature in quarantine (Q) and post-quarantine (PQ) settings. Data is aggregated across participants from all batches (n = 88). We asked participants to report, using a forced-choice “Yes/No” question, if they experienced any stressful event prior to answering the survey. If participants reported having experienced a stressful event, they could further decide to report the nature of the event by selecting one option from the eight-options list. (Top) The number of surveys in which participants did not report experience of stressful events were higher than those in which they reported occurrence of stressful events. (Bottom) Zooming on data which represents only the presence of stressful events helps to highlight the different nature of such events.

# **Figure S3:** Routine EMA survey responses to the question ‘Who are you interacting with right now?’. Participants chose one of the following options to answer the question: ‘No, I am alone’ (Alone), ‘Someone not so close to me’ (Distant), ‘Someone close to me’ (Close). Data is aggregated across all participants (n = 88). Q: quarantine, PQ: post-quarantine.

# **Figure S4**: Jittered plot showing responses to the question ‘How happy/joyful are you feeling right now?’ (positive affect) plotted against the responses to ‘How anxious/nervous/worried are you feeling right now?’ (negative affect). Data is aggregated across all participants (*n* = 88). Q: quarantine, PQ: post-quarantine.

# **Figure S5:** Histogram showing counts of different responses to the question ‘How happy/joyful are you feeling right now?’ (positive affect) and ‘How anxious/nervous/worried are you feeling right now?’ (negative affect). Data is aggregated across all participants (n = 88). Q: quarantine, PQ: post-quarantine.

# **Figure S6**: Estimates of fixed effects from fitted cumulative link mixed models (CLMMs) with random intercepts fitted to the ratings of positive and negative affect *: *p* < 0.5, ***: *p <* 0.001. The estimates and corresponding 95% Wald confidence intervals displayed here are reported in Table S8 (for positive affect) and Table S9 (for negative affect).

# **Figure S7**: Block-wise participant-level average trial duration in the patch-foraging task (*n* = 76). We considered trials from complete as well as incomplete patches to calculate average trial durations for each participant. Each black-colored diamond represents sample-level mean of individual-level average trial durations for the corresponding block; error bars show values at sample-level mean $\pm$ 2 $\times$ SD, where SD is the standard deviation in individual-level average trial durations. Participants were excluded from analysis if their average trial duration was greater than sample-level mean + 2 $\times$ SD in at least one block; data points belonging to such individuals (*n* = 10) are labelled with their arbitrarily assigned IDs in blue color. The cut-off for exclusion was 6.42 s and 5.59 s for the ‘short’ block (travel time = 3 s) and the ‘long’ block (travel time = 10 s), respectively.

# **Figure S8**: Jittered plot showing all trial durations from the patch-foraging task played by each participant who were excluded from analysis (*n = 10*). These participants’ average trial duration exceeded the limit of *sample-level mean trial duration + 2* $\times$ *SD* in either one or both blocks of the patch-foraging task. Trial durations exceeding the limit (for the corresponding block) are highlighted in red. Numbers following 'EP #' are arbitrarily assigned IDs (see Figure S7). EP: excluded participant.

# **Figure S9**: Jittered plot showing data of excluded participants who used extreme decision-making in the patch-foraging task. The panel titles display arbitrarily assigned IDs to participants who were excluded from analysis (*n = 6*). We could only estimate patch-level exit threshold if a participant made at least two harvests in the corresponding complete patch. However, the excluded participants shown here made at most one harvest in all the complete patches in one of the blocks played e.g., one participant (EP #D-5) made no harvests in any patch of the ‘short’ block (travel time = 3 s), and another participant (EP #h-1) made no harvests in the complete patches of the ‘long’ block (travel time = 10 s). Characters preceeding 'EP #' were arbitrarily assigned IDs to the participants. EP: excluded participant.

# **Figure S10**: Bounded nature of patch-level exit threshold. We estimated patch-level exit threshold ($\mu_{exit}$) by averaging the last two rewards ($r_{n-1}$ and $r_{n}$) participants received before they left the patch. Each patch in the task was initialized at a state of 7 units of reward (or berries); a Gaussian noise of $N$(0, 0.0625) was added to the state of the patch at each trial, and each harvest reduced the state of patch by 0.5: $r_{i}$ = max {0, 7 - 0.5 $\times$ $(n-1)+ N(0, \sigma=0.25)$} where $i$ indexed the trials in the patch, $i \epsilon$ {1, 2, …, *n, …, 25*}. (Left) We simulated the trajectory of $r_{i}$ for 5000 patches. (Right) For each (simulated) patch, we selected the maximum value of exit threshold and plotted the histogram: the maximum of simulated $\mu_{exit}$ across all patches was equal to 7.719 berries (red line).

# **Figure S11**: The estimated conditional modes of the random effects (and corresponding estimated variances) from the Beta generalized linear mixed-effects model (GLMM) with random intercepts only fitted to the data of block-level average exit thresholds (*n* = 60). (Left) Quantile-quantile (Q-Q) plot of estimated conditional modes of the random effects. (Right) Participant-specific random effects are visualised with error bars spanning $\pm$ 2 $\times$ conditional standard deviation around the estimated conditional modes. The tick labels of y-axis are arbitrarily assigned IDs to participants (ranging from 1 to 60); the labels alternate between left and right y-axes as we go from the lowest conditional mode to the highest conditional mode. The circles drawn with red and black borders follow similar pattern.

# **Figure S12**: Predictions for exit thresholds obtained for two participants using the Beta GLMM fitted to the patch-foraging task data of 60 participants. First, a reference grid was created using observed values of $NA_{unalike}$ ({0, 0.4, 0.5, 0.67, 0.83, 1} and travel time (3 s and 10 s) in the data. The predictor variables in the Beta GLMM, other than $NA_{unalike}$ and travel time, were set at their observed values for each participant: for the participant with highest estimated conditional mode of random effect, $PA_{unalike}$ = 0.5, $PA_{median}$ = 0, $NA_{median}$ = 0, (scaled) trait anxiety score = 0.567, and environment order = ‘shortLong’. Similarly, for the participant with lowest estimated conditional mode of random effect, $PA_{unalike}$ = 1, $PA_{median}$ = 1.5, $NA_{median}$ = 0, (scaled) trait anxiety score = 0.317, and environment order = ‘shortLong’ (cf. Figure S11 to see estimated conditional modes of the random effects of these participants). Predictions were obtained using estimates of fixed- and random-effect coefficients from the model (see Tables S10 & Figure S11). Confidence bands around predictions represent 95% Wald confidence intervals. Red and blue represent predictions for exit thresholds of the ‘short’ and the ‘long’ block, respectively. RE: random effect.

# **Figure S13:** Foraging results are robust to the exclusion of imputed exit-day cases. Estimates of Beta GLMMs fitted to datasets generated without considering quarantine exit information of participants vs. datasets generated using participants who reported quarantine exit day. Specifically, quarantine exit reporting was used to filter participants after applying exclusion criteria 1-3 mentioned in Supplementary Information Section 3.3. The direction and magnitude of key effects are similar across datasets. 're': estimated conditional modes of random effects from Beta GLMM. 'Exit R': quarantine exit day was reported. 'Exit R/E/NA': quarantine exit day was either reported, estimated, or not available. n = Number of participants in the dataset generated.

# **Figure S14**: EMA interaction outcomes are robust to exclusion of imputed cases. Results for (a) 'need to interact' and (b) 'type of interaction' remain consistent when analyses are restricted to participants with reported exit day. Model estimates are reported in Tables S6 and S7 for (a) and (b), respectively. 'Exit R': quarantine exit day was reported. 'Exit R/E': quarantine exit day was either reported or estimated. n = Number of participants in the dataset generated.

# **Figure S15:** Positive affect results are robust to exclusion of imputed cases. Estimates of positive affect are similar when analyses are restricted to participants with reported exit day. Model estimates are reported in Table S8.

# **Figure S16:** Negative affect results are robust to exclusion of imputed cases. Estimates of negative affect are consistent across datasets with and without imputed exit-day cases. Model estimates are reported in Table S9.

# **Figure S17:** Estimates of Beta GLMMs fitted to datasets generated under different exclusion criteria. The effect of travel time remains stable across all specifications, whereas the association of exit-threshold with variability in negative affect varies in magnitude and statistical significance.

# **Figure S18:** Completed routine EMA surveys (blue circles) and task submission time (green triangles) across different participants. Data shown belongs to the participants who made $\geq$ 10 decisions in each block of the first-instance of the task. Routine EMA surveys submitted outside the pre-game window of 48 hrs are not shown. A reference date of 31 December, 2021 was used to align task submission day while keeping the task submission time (hour-min-sec) unchanged for visualisation; *iDay* thus denotes the temporally aligned day of the referenced week. Distribution of EMA survey submission relative to task completion is shown to illustrate variability in temporal proximity between affect measurement and behavior.

# **Figure S19:** Task-day EMA reports (purple circles) used for estimating affect-related regressor values when using the ‘task-day EMA aggregation window’ in the analysis of patch-foraging task data. Data shown belongs to the participants who made $\geq$ 10 decisions in each block of the first-instance of the task. Routine EMA surveys submitted outside the pre-game window of 48 hrs are not shown.

# **Figure S20**: 95% confiderence intervals around the estimated effect of variability in negative affect on exit thresholds across datasets of the first-instance of the patch-foraging task generated under different pre-game aggregation windows and minimum trial counts. Effects are observed primarily when variability is computed using temporally proximal affect measures. Error bars not aligning with x-axis ticks (shown in brown) were shifted horizontally to prevent overlap. X-axis is not a linear scale, and tick labels display only the observed number of participants in the generated datasets.

# **Figure S21:** Linear mixed-effects model does not adequately summarize patch stay duration data. Deviation of DHARMa residuals from the expected, uniform distribution reveal clear model misspecification. DHARMa residuals (blue triangles) were generated using 1000 unconditional simulations from the fitted model. Black lines denote 95% CI around the expected location (red line) for DHARMa residuals obtained from a correctly specified model. KS test: (exact one-sample) Kolmogorov-Smirnov test.

# **Figure S22:** Simulated datasets (grey) from the linear mixed-effects model (LMM) do not resemble the observed patch stay duration data (red), indicating that the LMM is not appropriate to summarize the observed data. Simulations used estimated conditional modes of random effects.

# **Figure S23:** Disagreement of simulated data with observed (log-transformed) patch stay duration. Comparisons of summary statistics (minimum [left], median [middle], maximum [right]) between observed and simulated data demonstrate that the linear mixed-effects model does not adequately summarise (log-transformed) patch-stay duration. Test statistic *p* denotes the fraction of simulations (out of 1000) where the summary statistic for the simulated dataset was greater than or equal to the observed statistic.

# **Figure S24:** Gamma generalized linear mixed-model (GLMM) provides an adequate fit to the data of block-level average patch stay duration (n = 60). DHARMa residual diagnostics for the model show no systematic deviations from expected distributional properties, indicating that the model appropriately summarizes the data - we have only shown QQ plot of DHARMa residuals plotted against standard uniform distribution. Black lines denote 95% CI around the expected location (red line) for DHARMa residuals obtained from a correctly specified model. KS test: (exact one-sample) Kolmogorov-Smirnov test.n.s. = not significant.

# **Figure S25:** Posterior predictive checks confirm good fit of the Gamma GLMM fitted to block-level average patch stay duration. Observed averages (red) fall within the range of simulated datasets (grey) generated from the fitted model, demonstrating that the model captures both central tendency and variability of the observed data.

# **Figure S26:** Patch stay duration increases with travel time. Block-level average stay duration is higher in the block with long travel-time (= 10 s) compared to the block with short travel-time (= 3 s), consistent with theoretical predictions from Marginal Value Theorem and replicating the pattern observed using exit-threshold measures. n = Number of participants.

# **Figure S27**: Associations of key predictors with block-level average patch stay duration are similar across different estimation procedures used to fit Gamma GLMMs and Gamma GLM (Base R). Estimates from fitted models show a positive effect of travel time on average stay duration, and a similar pattern of association with variability in negative affect as observed in exit-threshold analyses. AGQ = Adaptive Gauss-Hermite Quadrature method.

# **Table S1:** Questions asked in the routine EMA surveys and their possible responses.

| **No.** | **Question** | **Possible responses** |
| --- | --- | --- |
| 1 | Who are you interacting with right now? | 0 - No, I am alone.  1 - Someone not as close to me  2 - Someone close to me |
| 2 | Are you engaged in a face-to-face conversation?^⌗^ | 0 - No  1 - Yes |
| 3 | Are you engaged in real-time digital (phone text video) conversation?^⌗^ | 0 - No  1 - Yes |
| 4 | Do you want to be with other people right now?^⌗^ | 0 - Not at all  1 - Maybe  2 - Slightly  3 - Definitely  4 - Most definitely |
| 5 | Did you take a nap since the last survey? | 0 - No  1 - Yes |
| 6 | How was your nap? | 0 - Very poor  1 - Poor  2 - Okay  3 - Good  4 - Very good |
| 7 | Did anything stressful event occur since the last survey?^&^ A stressful event is any event, even a minor one which negatively affects you. | 0 - No  1 - Yes |
| 8 | How would you describe this stressful event?(You can choose more than one category to describe the event.)^!^ | 0 - Argument/conflict/disagreement  1 - Financial event  2 - Home-related event  3 - Work-related event  4 - Health event(including COVID)  5 - Event that happened to others  6 - Travel-related event  7 - Other  List format |
| 9 | When did this event happen?^⌗&^ | 0 - 0-30 minutes ago  1 - 3-60 minutes ago  2 - 60+ minutes ago |
| 10 | How unpleasant was this event on a scale of 0 to 100?(0 means not at all stressful whereas 100 means extremely stressful.)^⌗^ | Integer (0-100) |
| 11 | Did any positive event happen since the last survey?(A positive/pleasant event is any event even a minor one which improves your mood or make you feel good.)^⌗^ | 0 - No  1 - Yes |
| 12 | How would you describe this pleasant event? (You can choose more than one category to describe the event.)^⌗^ | 0 - Agreement/feedback/discussion - related event  1 - Financial event  2 - Home-related event  3 - Work-related event  4 - Health event(including COVID)  5 - Event that happened to others  6 - Travel-related event  7 - Other |
| 13 | When did this positive event occur?^⌗^ | 0 - 0-30 minutes ago  1 - 3-60 minutes ago  2 - 60+ minutes ago |
| 14 | How pleasant was this event on a scale of 0 to 100?(0 means not all pleasant whereas 100 means extremely pleasant.)^⌗^ | Integer (0-100) |
| 15 | What are you experiencing right now?(You can select more than one option.) | 0 - Happiness  1 - Sadness  2 - Fear  3 - Anger  4 - Disgust  5 - Surprise  6 - Neutral |
| 16 | How happy/joyful/cheerful are you feeling at the moment? | 0 - Not at all  1 - Slightly  2 - Moderately  3 - Very  4 - Extremely |
| 17 | How anxious/worried/nervous are you feeling at the moment? | 0 - Not at all  1 - Slightly  2 - Moderately  3 - Very  4 - Extremely |

*Note.* The integers preceding the responses were used to encode the responses for analysis; the integers were not shown to the participants. [^⌗^: Participants could skip the question if they wanted to. ^&^: The question had unintended grammatical error(s). ^!^: Participants could choose only one of the responses.]

# **Table S2**: Overview of quarantine-exit survey scheduling across batches.

| **Batch** | **Arrival date (year of 2021)** | **Survey date** | **Number of participants** | **Survey time and active period** |
| --- | --- | --- | --- | --- |
| B1 | July 19 | July 24 | 10 | 3 PM;  no expiry for survey |
|  | July 20 |  | 10 |  |
| B2 | July 29 | August 3 | 12 | 3 PM;  no expiry for survey |
|  | July 30 |  | 12 |  |
| B3 | August 9 | August 14 | 7 | 3 PM;  no expiry for survey |
|  | August 10 |  | 9 |  |
| B4 | August 19 | August 24 | 4 | 4:30 PM;  no expiry for survey |
|  | August 20 |  | 5 |  |
| B5 | August 31 | September 6 | 13 | 5 PM;  no expiry for survey |
|  | September 1 |  | 10 |  |
|  | September 2 |  | 5 |  |
| B6 | September 9 | September 14 | 8 | 4:30 PM;  no expiry for survey |
|  | September 10 |  | 6 |  |
| B7 | October 18 | October 22 | 2 | 4 PM;  no expiry for survey |
|  | October 19 |  | 3 |  |

*Note.* For logistical purposes, IIT Kanpur administration had asked students from each batch to return on campus on different but adjacent dates. These different arrival dates of participants in our study belonging to the same batch are shown here. The institute had informed students returning to the campus that they would be tested for COVID-19 following three days in quarantine and be allowed to move in their residential hostels on the following day if tested negative e.g., for students arriving on campus on July 20 (Day Zero), the institute had planned to administer the test on July 23 (Day Three) and expected testing centers to generate test results by the morning of the next day (Day Four). Thus, we sent a quarantine survey to the participants in the afternoon of Day Four (except for a few participants in Batch B7, who received a quarantine survey on Day Three).

# **Table S3**: Overview of different test day - exit day combinations reported by the participants in quarantine-exit survey.

| **Test day (actual or expected)** | **Exit day (actual or expected)** | **Number of participants** | **Percentage (of total participants)** |
| --- | --- | --- | --- |
| 2 | 3 | 2 | 1.72 |
| 2 | 4 | 1 | 0.86 |
| 3 | 3 | 1 | 0.86 |
| 3 | 4 | 82 | 70.7 |
| 3 | 5 | 3 | 2.59 |
| 3 | *Did not report* | 7 | 6.03 |
| 4 | 5 | 3 | 2.59 |
| 4 | 9 | 1 | 0.86 |
| 4 | *Did not report* | 1 | 0.86 |
| 5 | *5* | 2 | 1.72 |
| *Did not repor*t | *Did not report* | 13 | 11.2 |
| Total participants: | | 116 | 100 |

*Note.* Three rows have been highlighted with pale blue to display the number of participants who did not report exit day from quarantine (*n = 21*). We removed one participant (highlighted with red) from further analysis as the duration between their exit day and test day was equal to 5 days which did not seem possible in the context of the study given that they had indicated their COVID-19 test was negative.

# **Table S4:** Approximating quarantine-exit day using batch-wise data.

| **Index No.** | **Batch** | **Test day** | **Exit day** | **No. of**  **participants** | **Estimated**  **exit day** |
| --- | --- | --- | --- | --- | --- |
| 1 | B1 | *Did not report* | *Did not repor*t | 3 | - |
| 2 | B1 | 3 | 4 | 15 | NN |
| 3 | B1 | 3 | 5 | 2 | NN |
| 4 | B2 | *Did not report* | *Did not report* | 2 | 4 |
| 5 | B2 | 3 | 4 | 22 | NN |
| 6 | B3 | *Did not report* | *Did not report* | 1 | - |
| 7 | B3 | 2 | 3 | 1 | NN |
| 8 | B3 | 2 | 4 | 1 | NN |
| 9 | B3 | 3 | 4 | 13 | NN |
| 10 | B4 | *Did not report* | *Did not report* | 2 | - |
| 11 | B4 | 3 | 4 | 3 | NN |
| 12 | B4 | 4 | 5 | 2 | NN |
| 13 | B4 | 5 | 5 | 2 | NN |
| 14 | B5 | *Did not report* | *Did not report* | 3 | - |
| 15 | B5 | 2 | 3 | 1 | NN |
| 16 | B5 | 3 | *Did not report* | 5 | 5 |
| 17 | B5 | 3 | 3 | 1 | NN |
| 18 | B5 | 3 | 4 | 14 | NN |
| 19 | B5 | 3 | 5 | 1 | NN |
| 20 | B5 | 4 | *Did not report* | 1 | 5 |
| 21 | B5 | 4 | 5 | 1 | NN |
| 22 | B6 | *Did not report* | *Did not report* | 2 | 4 |
| 23 | B6 | 3 | 4 | 12 | NN |
| 24 | B7 | 3 | *Did not report* | 2 | 4 |
| 25 | B7 | 3 | 4 | 3 | NN |
| Total participants: | | | | 115 |  |

*Note.* For participants who reported only the test day, we approximated their exit day from quarantine with the latest exit day reported by other participants in the corresponding batch (see Index No. 20, for example). If participants did not report the test day, we approximated their exit day only if all other participants in their batch reported the same quarantine exit day (see, for example, the rows of B6). No approximation was done for participants whose batch did not report a common exit day if they did not report their test day (see Index No. 1 or Index No. 14, for example). Similarly, no approximation was needed for participants who reported the day of their quarantine exit. NN: Not needed.

# **Table S5:** Overview of different test day - exit day combinations of participants analysed in the study.

| **Test day** | **Exit day status** | **Exit day** | **Number of participants** | **Percentage (of total participants)** |
| --- | --- | --- | --- | --- |
| 2 | Reported | 3 | 1 | 1.14 |
|  | Reported | 4 | 1 | 1.14 |
| 3 | Reported | 4 | 70 | 79.55 |
|  | Reported | 5 | 3 | 3.41 |
| 4 | Reported | 5 | 3 | 3.41 |
| 5 | Reported | 5 | 2 | 2.27 |
| **Total:** | | | **80** | **90.91** |
| 3 | Estimated | 4 | 4 | 4.55 |
|  | Estimated | 5 | 2 | 2.27 |
| 4 | Estimated | 5 | 1 | 1.37 |
| Not reported | Estimated | 4 | 1 | 1.14 |
| **Total:** | | | **88** | **100** |

# **Table S6**: Estimated fixed-effect coefficients from cumulative link mixed models with ‘Interaction-intensity’ as the response variable.

| **Data & model**  **Term** | **Exit_R/E_**  **~ TA + SC +**  **(1 \| ID)** | **Exit_R_**  **~ TA + SC +**  **(1 \| ID)** | **Exit_R/E_**  **~ TA + SC +**  **(1 + SC \| ID)** | **Exit_R_**  **~ TA + SC +**  **(1 + SC \| ID)** | **Exit_R/E_**  **~ TA * SC +**  **(1 \| ID)** | **Exit_R_**  **~ TA * SC +**  **(1 \| ID)** |
| --- | --- | --- | --- | --- | --- | --- |
| 0\|1 | -0.096 (p = 0.871) [-1.248, 1.056] | 0.011 (p = 0.986) [-1.180, 1.201] | -0.087 (p = 0.887 )  [-1.287, 1.114] | 0.025 (p = 0.968)  [-1.211, 1.261] | 0.028 (p = 0.963)  [-1.148, 1.203] | 0.131 (p = 0.833)  [-1.084, 1.346] |
| 1\|2 | 1.443 (p = 0.014) [0.289, 2.596] | 1.537 (p = 0.012) [0.344, 2.729] | 1.544 (p = 0.012)  [0.342, 2.747] | 1.645 (p = 0.009) [0.407, 2.884] | 1.567 (p = 0.009) [0.390, 2.745] | 1.658 (p = 0.008) [0.440, 2.876] |
| 2\|3 | 2.624 (p = 0.000) [1.467, 3.781] | 2.625 (p = 0.000) [1.429, 3.821] | 2.803 (p = 0.000)  [1.597, 4.01] | 2.810 (p = 0.000) [1.568, 4.053] | 2.749 (p = 0.000) [1.568, 3.930] | 2.747 (p = 0.000) [1.526, 3.968] |
| 3\|4 | 4.420 (p = 0.000) [3.248, 5.593] | 4.486 (p = 0.000) [3.273, 5.698] | 4.733 (p = 0.000)  [3.509, 5.957] | 4.806 (p = 0.000) [3.545, 6.068] | 4.546 (p = 0.000) [3.349, 5.742] | 4.607 (p = 0.000) [3.369, 5.844] |
| TA | 2.430 (p = 0.054) [-0.042, 4.902] | 2.498 (p = 0.054) [-0.043, 5.040] | 2.682 (p = 0.042)  [0.103, 5.261] | 2.771 (p = 0.040) [0.128, 5.413] | 2.718 (p = 0.035) [0.187, 5.249] | 2.776 (p = 0.037) [0.173, 5.379] |
| SC [PQ - Q] | -0.526 (p = 0.000) [-0.705, -0.348] | -0.491 (p = 0.000) [-0.679, -0.304] | -0.655 (p = 0.000)  [-1.000, -0.310] | -0.634 (p=0.001)  [-1.002, -0.265] | -0.280 (p = 0.265)  [-0.772, 0.212] | -0.258 (p = 0.318)  [-0.764, 0.248] |
| TA ×  SC [PQ] | - | - | - | - | -0.569 (p = 0.293)  [-1.629, 0.492] | -0.537 (p = 0.331)  [-1.619, 0.545] |
| SD  (random slopes) | - | - | 1.325 | 1.350 | - | - |
| Num.Obs. | 1983 | 1807 | 1983 | 1807 | 1983 | 1807 |
| R^2^ Marg. | 0.034 | 0.035 | 0.038 | 0.040 | 0.034 | 0.035 |
| R^2^ Cond. | 0.558 | 0.560 | 0.617 | 0.622 | 0.558 | 0.560 |
| AIC | 4791.7 | 4348.8 | 4722.3 | 4285.3 | 4792.6 | 4349.9 |
| BIC | 4830.9 | 4387.3 | 4772.7 | 4334.8 | 4837.4 | 4393.9 |

*Note*. The first four terms represent thresholds (or cut-points) where each integer encodes one of the five possible answers to the question ‘Do you want to be with other people right now?’: 0 = Not at all, 1 = Maybe, 2 = Slightly, 3 = Definitely, 4 = Most definitely. Values in square brackets denote 95% confidence interval around the estimated coefficient. Exit_R/E_: dataset generated using reported or estimated quarantine exit day (number of participants = 88), Exit_R_: dataset generated using only reported quarantine exit day (number of participants = 80), TA: min-max scaled trait anxiety score, PQ: post-quarantine, SD: standard deviation.

# **Table S7:** Estimated fixed-effect coefficients from cumulative link mixed models with ‘Interaction-with’ as the response variable.

| **Data & model**  **Term** | **Exit_R/E_**  **~ TA + SC +**  **(1 \| ID)** | **Exit_R_**  **~ TA + SC +**  **(1 \| ID)** | **Exit_R/E_**  **~ TA + SC +**  **(1 + SC \| ID)** | **Exit_R_**  **~ TA + SC +**  **(1 + SC \| ID)** | **Exit_R/E_**  **~ TA * SC +**  **(1 \| ID)** | **Exit_R_**  **~ TA * SC +**  **(1 \| ID)** |
| --- | --- | --- | --- | --- | --- | --- |
| 0\|1 | 0.088 (p = 0.806)  [-0.612, 0.787] | 0.102 (p = 0.774)  [-0.595, 0.799] | 0.141 (p = 0.697)  [-0.570, 0.852] | 0.135 (p = 0.711)  [-0.579, 0.849] | 0.130 (p = 0.737)  [-0.627, 0.886] | 0.179 (p = 0.644)  [-0.580, 0.938] |
| 1\|2 | 0.971 (p = 0.007) [0.270, 1.672] | 0.929 (p = 0.009) [0.230, 1.628] | 1.042 (p = 0.004) [0.329, 1.754] | 0.977 (p = 0.007) [0.261, 1.693] | 1.013 (p = 0.009) [0.255, 1.771] | 1.006 (p = 0.010) [0.245, 1.766] |
| TA | -1.602 (p = 0.037)  [-3.107, -0.098] | -1.545 (p =0.042)  [-3.036, -0.055] | -1.662 (p = 0.025)  [-3.118, -0.206] | -1.622 (p = 0.030)  [-3.086, -0.159] | -1.502 (p = 0.075)  [-3.157, 0.153] | -1.362 (p = 0.106)  [-3.013, 0.289] |
| SC [PQ - Q] | 0.258 (p = 0.010) [0.061, 0.455] | 0.264 (p = 0.012) [0.059, 0.468] | 0.365 (p = 0.008) [0.096, 0.635] | 0.349 (p = 0.013) [0.074, 0.624] | 0.332 (p = 0.230)  [-0.210, 0.873] | 0.396 (p = 0.162)  [-0.159, 0.950] |
| TA × SC [PQ] | - | - | - | - | -0.176 (p = 0.776)  [-1.387, 1.036] | -0.316 (p = 0.615)  [-1.548, 0.916] |
| SD  (random slope) | - | - | 0.724 | 0.682 | - | - |
| Num. Obs. | 1984 | 1808 | 1984 | 1808 | 1984 | 1808 |
| R^2^ Marg. | 0.021 | 0.021 | 0.025 | 0.025 | 0.021 | 0.021 |
| R^2^ Cond. | 0.288 | 0.273 | 0.319 | 0.302 | 0.287 | 0.272 |
| AIC | 3369.9 | 3098.5 | 3361.9 | 3094.2 | 3371.8 | 3100.2 |
| BIC | 3397.9 | 3126.0 | 3401.1 | 3132.7 | 3405.4 | 3133.2 |

*Note.* The first two terms represent thresholds (or cut-points) where each integer encodes one of the three possible answers to the question ‘Whom are you interacting with right now?’: 0 = No, I am alone [Alone], 1 = Someone not so close to me [Distant], 2 = Someone close to me [Close]. Values in square brackets denote 95% confidence interval aroud the estimated coefficient. Exit_R/E_: dataset generated using reported or estimated quarantine exit day (number of participants = 88), Exit_R_: dataset generated using only reported quarantine exit day (number of participants = 80), TA: min-max scaled trait anxiety score, PQ: post-quarantine, SD: standard deviation.

# **Table S8**: Estimated fixed-effect coefficients from cumulative link mixed models fitted to the data of positive affect.

| **Data & model**  **Term** | **Exit_R/E_**  **~ TA * SC * IW +**  **(1 \| ID)** | **Exit_R_**  **~ TA * SC * IW +**  **(1 \| ID)** | **Exit_R/E_**  **~ TA * SC * IW +**  **(1 + SC \| ID)** | **Exit_R_**  **~ TA * SC * IW +**  **(1 + SC \| ID)** |
| --- | --- | --- | --- | --- |
| 0\|1 | -2.518 (p = 0.000)  [-3.401, -1.635] | -2.256 (p = 0.000)  [-3.138, -1.373] | -2.508 (p = 0.000)  [-3.404, -1.612] | -2.221 (p = 0.000)  [-3.118, -1.325] |
| 1\|2 | -0.586 (p = 0.190)  [-1.462, 0.290] | -0.378 (p = 0.397)  [-1.254, 0.498] | -0.499 (p = 0.271)  [-1.387, 0.390] | -0.272 (p = 0.550)  [-1.162, 0.619] |
| 2\|3 | 1.894 (p = 0.000)  [1.011, 2.778] | 1.985 (p = 0.000)  [1.099, 2.870] | 2.051 (p = 0.000)  [1.152, 2.950] | 2.158 (p = 0.000) [1.255, 3.060] |
| 3\|4 | 4.184 (p = 0.000)  [3.216, 5.152] | 4.193 (p = 0.000)  [3.222, 5.163] | 4.409 (p = 0.000)  [3.420, 5.398] | 4.436 (p = 0.000) [3.443, 5.430] |
| TA | -3.642 (p = 0.000)  [-5.520, -1.764] | -3.346 (p = 0.000)  [-5.214, -1.477] | -3.612 (p = 0.000)  [-5.510, -1.713] | -3.301 (p = 0.001)  [-5.194, -1.409] |
| SC [PQ - Q] | -0.542 (p = 0.077)  [-1.143, 0.058] | -0.520 (p = 0.094)  [-1.128, 0.088] | -0.410 (p = 0.330)  [-1.234, 0.415] | -0.361 (p = 0.398)  [-1.198, 0.476] |
| InteractWith [Distant] | 0.411 (p = 0.520)  [-0.842, 1.664] | 0.581 (p = 0.398)  [-0.767, 1.928] | 0.342 (p = 0.603)  [-0.948, 1.633] | 0.561 (p = 0.429)  [-0.830, 1.953] |
| InteractWith [Close] | 0.072 (p = 0.880)  [-0.867, 1.011] | 0.238 (p = 0.621)  [-0.707, 1.183] | 0.217 (p = 0.669)  [-0.776, 1.211] | 0.413 (p = 0.417)  [-0.585, 1.410] |
| TA × SC [PQ] | 0.577 (p = 0.368)  [-0.678, 1.831] | 0.475 (p = 0.464)  [-0.796, 1.746] | 0.372 (p = 0.675)  [-1.370, 2.115] | 0.269 (p = 0.765)  [-1.496, 2.035] |
| TA × InteractWith [Distant] | -0.682 (p = 0.667)  [-3.785, 2.421] | -1.148 (p = 0.492)  [-4.428, 2.131] | -0.119 (p = 0.942)  [-3.316, 3.078] | -0.657 (p = 0.703)  [-4.041, 2.726] |
| TA × InteractWith [Close] | 1.692 (p = 0.104)  [-0.346, 3.731] | 1.356 (p = 0.193)  [-0.685, 3.397] | 1.704 (p = 0.119)  [-0.438, 3.847] | 1.346 (p = 0.218)  [-0.796, 3.487] |

| **Data & model**  **Term** | **Exit_R/E_**  **~ TA * SC * IW +**  **(1 \| ID)** | **Exit_R_**  **~ TA * SC * IW +**  **(1 \| ID)** | **Exit_R/E_**  **~ TA * SC * IW +**  **(1 + SC \| ID)** | **Exit_R_**  **~ TA * SC * IW +**  **(1 + SC \| ID)** |
| --- | --- | --- | --- | --- |
| SC [PQ] × InteractWith [Distant] | 0.489 (p = 0.515)  [-0.985, 1.963] | 0.467 (p = 0.559)  [-1.098, 2.033] | 0.716 (p = 0.371)  [-0.854, 2.287] | 0.623 (p = 0.463)  [-1.042, 2.289] |
| SC [PQ] × InteractWith [Close] | 1.429 (p = 0.024)  [0.191, 2.668] | 1.292 (p = 0.041)  [0.053, 2.532] | 1.289 (p = 0.058)  [-0.045, 2.623] | 1.118 (p = 0.100)  [-0.214, 2.451] |
| TA × SC [PQ] × InteractWith [Distant] | 0.543 (p = 0.768)  [-3.064, 4.149] | 0.837 (p = 0.664)  [-2.939, 4.612] | -0.461 (p = 0.812)  [-4.260, 3.339] | -0.100 (p = 0.961)  [-4.082, 3.881] |
| TA × SC [PQ] × InteractWith [Close] | -1.979 (p = 0.153)  [-4.693, 0.735] | -1.674 (p = 0.227)  [-4.388, 1.040] | -2.162 (p = 0.146)  [-5.077, 0.753] | -1.877 (p = 0.206)  [-4.788, 1.033] |
| SD (random slopes for SC) | - | - | 0.929 | 0.921 |
| Number of observations | 1984 | 1808 | 1984 | 1808 |
| R^2^ Marginal | 0.106 | 0.108 | 0.105 | 0.106 |
| R^2^ Conditional | 0.427 | 0.414 | 0.463 | 0.450 |
| AIC | 4526.8 | 4150.5 | 4496.4 | 4124.8 |
| BIC | 4616.3 | 4238.5 | 4597.1 | 4223.8 |

Note. The first four terms in the ‘Term’ column represent threshold parameters (or cut-points) of CLMMs, where each integer encodes one of the five possible responses to the question ‘How happy/joyful/cheerful are you feeling right now?’: 0 = Not at all, 1 = Slightly, 2 = Moderately, 3 = Very, 4 = Extremely. ‘SC’ refers to social context and was encoded as a categorical variable with two distinct levels: quarantine (Q) and post-quarantine (PQ) . The term ‘InteractionWith’ was encoded as a categorical variable with three distinct levels: Alone, Distant, Close. Values in square brackets denote 95% confidence interval aroud the estimated coefficient. Exit_R/E_: dataset generated using reported or estimated quarantine exit day (number of participants = 88), Exit_R_: dataset generated using only reported quarantine exit day (number of participants = 80), TA: min-max scaled trait anxiety score, PQ: post-quarantine, SD: standard deviation.

# **Table S9:** Estimated fixed-effect coefficients from cumulative link mixed models fitted to the data of negative affect.

| **Data & model**  **Term** | **Exit_R/E_**  **~ TA * SC * IW +**  **(1 \| ID)** | **Exit_R_**  **~ TA * SC * IW +**  **(1 \| ID)** | **Exit_R/E_**  **~ TA * SC * IW +**  **(1 + SC \| ID)** | **Exit_R_**  **~ TA * SC * IW + (1 + SC \| ID)** |
| --- | --- | --- | --- | --- |
| 0\|1 | 2.725 (p = 0.000)  [1.612, 3.838] | 2.777 (p = 0.000)  [1.664, 3.890] | 2.788 (p = 0.000)  [1.573, 4.003] | 2.841 (p = 0.000) [1.614, 4.068] |
| 1\|2 | 4.621 (p = 0.000)  [3.495, 5.747] | 4.663 (p = 0.000)  [3.536, 5.790] | 4.762 (p = 0.000)  [3.534, 5.990] | 4.812 (p = 0.000) [3.570, 6.054] |
| 2\|3 | 6.730 (p = 0.000)  [5.572, 7.888] | 6.622 (p = 0.000)  [5.460, 7.784] | 6.927 (p = 0.000)  [5.667, 8.187] | 6.827 (p = 0.000) [5.551, 8.103] |
| 3\|4 | 8.247 (p = 0.000)  [7.039, 9.455] | 8.170 (p = 0.000)  [6.953, 9.388] | 8.478 (p = 0.000)  [7.170, 9.786] | 8.412 (p = 0.000) [7.082, 9.742] |
| TA | 5.860 (p = 0.000)  [3.520, 8.199] | 5.839 (p = 0.000)  [3.520, 8.158] | 5.997 (p = 0.000)  [3.449, 8.545] | 5.980 (p = 0.000) [3.425, 8.534] |
| SC [PQ - Q] | -0.530 (p = 0.154)  [-1.259, 0.199] | -0.541 (p = 0.158)  [-1.291, 0.209] | -0.585 (p = 0.256)  [-1.594, 0.424] | -0.606 (p = 0.258) [-1.658, 0.445] |
| InteractWith [Distant] | -0.065 (p = 0.931)  [-1.533, 1.404] | -0.456 (p = 0.578)  [-2.063, 1.151] | 0.041 (p = 0.958)  [-1.474, 1.556] | -0.436 (p = 0.608) [-2.101, 1.230] |
| InteractWith [Close] | -0.499 (p = 0.405)  [-1.672, 0.675] | -0.567 (p = 0.348)  [-1.751, 0.617] | -0.736 (p = 0.255)  [-2.006, 0.533] | -0.844 (p = 0.200) [-2.137, 0.448] |
| TA × SC [PQ] | 0.917 (p = 0.200)  [-0.487, 2.321] | 0.918 (p = 0.207)  [-0.508, 2.344] | 1.070 (p = 0.290)  [-0.913, 3.052] | 1.085 (p = 0.301)  [-0.973, 3.144] |
| TA × InteractWith [Distant] | 0.323 (p = 0.845)  [-2.915, 3.561] | 1.149 (p = 0.513)  [-2.291, 4.590] | -0.064 (p = 0.970)  [-3.422, 3.294] | 0.925 (p = 0.613)  [-2.657, 4.506] |
| TA × InteractWith [Close] | 0.907 (p = 0.468)  [-1.543, 3.358] | 1.034 (p = 0.409)  [-1.420, 3.488] | 1.420 (p = 0.292)  [-1.222, 4.062] | 1.642 (p = 0.228)  [-1.026, 4.311] |

| **Data & model**  **Term** | **Exit_R/E_**  **~ TA * SC * IW +**  **(1 \| ID)** | **Exit_R_**  **~ TA * SC * IW +**  **(1 \| ID)** | **Exit_R/E_**  **~ TA * SC * IW +**  **(1 + SC \| ID)** | **Exit_R_**  **~ TA * SC * IW + (1 + SC \| ID)** |
| --- | --- | --- | --- | --- |
| SC [PQ] × InteractWith [Distant] | -0.264 (p = 0.775)  [-2.079, 1.551] | 0.103 (p = 0.918)  [-1.849, 2.054] | -0.399 (p = 0.684)  [-2.317, 1.520] | 0.074 (p = 0.944)  [-1.993, 2.141] |
| SC [PQ] × InteractWith [Close] | 0.850 (p = 0.287)  [-0.713, 2.412] | 0.923 (p = 0.250)  [-0.651, 2.497] | 0.999 (p = 0.251)  [-0.709, 2.707] | 1.122 (p = 0.205)  [-0.611, 2.854] |
| TA × SC [PQ] ×  InteractWith [Distant] | 0.363 (p = 0.858)  [-3.624, 4.350] | -0.472 (p = 0.825)  [-4.659, 3.716] | 0.757 (p = 0.724)  [-3.449, 4.963] | -0.267 (p = 0.906) [-4.698, 4.165] |
| TA × SC [PQ] × InteractWith [Close] | -2.060 (p = 0.214)  [-5.310, 1.191] | -2.212 (p = 0.183)  [-5.465, 1.042] | -2.481 (p = 0.171)  [-6.031, 1.068] | -2.769 (p = 0.130) [-6.349, 0.811] |
| SD (random slopes for SC) |  |  | 0.977 | 1.024 |
| Number of observations | 1984 | 1808 | 1984 | 1808 |
| R^2^ Marginal | 0.171 | 0.185 | 0.173 | 0.187 |
| R^2^ Conditional | 0.558 | 0.550 | 0.589 | 0.584 |
| AIC | 3681.7 | 3341.9 | 3655.7 | 3314.8 |
| BIC | 3771.2 | 3429.9 | 3756.4 | 3413.8 |

Note. The first four terms in the ‘Term’ column represent threshold parameters (or cut-points) of CLMMs, where each integer encodes one of the five possible responses to the question ‘How anxious/worried/nervous are you feeling at the moment?’: 0 = Not at all, 1 = Slightly, 2 = Moderately, 3 = Very, 4 = Extremely. SC’ refers to social context and was encoded as a categorical variable with two distinct levels: quarantine (Q) and post-quarantine (PQ) . The term ‘InteractionWith’ was encoded as a categorical variable with three distinct levels: Alone, Distant, Close. Values in square brackets denote 95% confidence interval aroud the estimated coefficient. Exit_R/E_: dataset generated using reported or estimated quarantine exit day (number of participants = 88), Exit_R_: dataset generated using only reported quarantine exit day (number of participants = 80), TA: min-max scaled trait anxiety score, PQ: post-quarantine, SD: standard deviation.

# **Table S10**: Estimates of fixed-effect coefficients from the Beta generalized linear mixed model (GLMM) fitted to the data of doubly-transformed exit thresholds from the first-instance of the patch-foraging task (n = 60).

| Term | **Estimate** | **Standard error** | ***z*** | **p-value** | **95% CI** |
| --- | --- | --- | --- | --- | --- |
| Intercept | 0.819 | 0.348 | 2.352 | 0.019 | [0.136, 1.501] |
| Travel time  *[10-3]* | -0.508 | 0.099 | -5.161 | 0.000 | [-0.702, -0.315] |
| Environment order  [*shortLong-longShort*] | -0.507 | 0.212 | -2.394 | 0.017 | [-0.922, -0.092] |
| Scaled trait anxiety | 0.030 | 0.593 | 0.051 | 0.959 | [-1.131, 1.192] |
| PA_unalike_ | 0.191 | 0.296 | 0.645 | 0.519 | [-0.388, 0.77] |
| NA_unalike_ | -1.070 | 0.298 | -3.588 | 0.000 | [-1.655, -0.486] |
| PA_median_ | -0.011 | 0.128 | -0.088 | 0.930 | [-0.262, 0.24] |
| NA_median_ | -0.031 | 0.135 | -0.227 | 0.820 | [-0.296, 0.234] |

*Note.* The Beta GLM was fitted with the R package *glmmTMB*. The standard deviation in

random effects were 0.622.

# **Table S11**: Marginal coefficients and their standard errors obtained from the Beta GLMM fitted to the data of the first-game instance of the patch-foraging task (*n* = 60).

| **Term** | **Estimate** | **Standard error** | ***z*** | **p-value** | **95% CI** |
| --- | --- | --- | --- | --- | --- |
| Intercept | 0.739 | 0.283 | 2.616 | 0.009 | [0.185, 1.293] |
| Travel time  ***[10-3]*** | -0.466 | 0.072 | -6.498 | 0.000 | [-0.607, -0.326] |
| Environment order  ***[shortLong-longShort]*** | -0.466 | 0.225 | -2.072 | 0.038 | [-0.906, -0.025] |
| Scaled trait anxiety | 0.040 | 0.528 | 0.075 | 0.940 | [-0.996, 1.075] |
| PA_unalike_ | 0.181 | 0.271 | 0.670 | 0.503 | [-0.349, 0.712] |
| NA_unalike_ | -0.981 | 0.263 | -3.733 | 0.000 | [-1.497, -0.466] |
| PA_median_ | -0.008 | 0.111 | -0.069 | 0.945 | [-0.225, 0.21] |
| NA_median_ | -0.026 | 0.126 | -0.203 | 0.839 | [-0.273, 0.221] |

*Note.* The Beta GLMM used to obtain marginal coeffiecients was fitted using the R package *GLMMadaptive* - its estimates were very similar to those of the Beta GLMM fitted using *glmmTMB* (see Table S10).

# **Table S12**: Estimated fixed-effect coefficients from the Beta GLMM fitted to the data of doubly-transformed exit thresholds of the non-influential participants (*n* = 56) from the first-game instance of the patch foraging task.

| **Term** | **Estimate** | **Standard error** | ***z*** | **p-value** | **95% CI** |
| --- | --- | --- | --- | --- | --- |
| Intercept | 0.880 | 0.251 | 3.508 | 0.000 | [0.388, 1.371] |
| Travel time  ***[10-3]*** | -0.500 | 0.096 | -5.223 | 0.000 | [-0.688, -0.313] |
| Environment order  ***[shortLong-longShort]*** | -0.341 | 0.154 | -2.213 | 0.027 | [-0.642, -0.039] |
| Scaled trait anxiety | -0.359 | 0.433 | -0.828 | 0.408 | [-1.208, 0.491] |
| PA_unalike_ | 0.108 | 0.219 | 0.491 | 0.623 | [-0.322, 0.538] |
| NA_unalike_ | -0.558 | 0.225 | -2.479 | 0.013 | [-0.999, -0.117] |
| PA_median_ | 0.065 | 0.092 | 0.711 | 0.477 | [-0.115, 0.245] |
| NA_median_ | -0.072 | 0.097 | -0.737 | 0.461 | [-0.262, 0.119] |

*Note.* The standard deviation in random effects was 0.366.

# **Table S13**: Estimates of fixed-effect coefficients from the Beta GLMM fitted to the data of (block-level) scaled exit thresholds from both-instances of the patch-foraging task (*n* = 32).

| **Term** | **Estimate** | **Standard error** | ***z*** | **p-value** | **95% CI** |
| --- | --- | --- | --- | --- | --- |
| Intercept | 0.768 | 0.394 | 1.951 | 0.051 | [-0.004, 1.539 ] |
| Travel time  ***[10-3]*** | -0.449 | 0.083 | -5.399 | 0.000 | [-0.612, -0.286] |
| Environment order  ***[shortLong-longShort]*** | -0.118 | 0.250 | -0.470 | 0.638 | [-0.608, 0.372] |
| Scaled trait anxiety | -0.359 | 0.776 | -0.462 | 0.644 | [-1.88, 1.162] |
| PA_unalike_ | -0.195 | 0.176 | -1.110 | 0.267 | [-0.54, 0.15 ] |
| NA_unalike_ | -0.438 | 0.200 | -2.191 | 0.028 | [-0.83, -0.046] |
| PA_median_ | -0.069 | 0.091 | -0.753 | 0.451 | [-0.247, 0.11] |
| NA_median_ | 0.134 | 0.101 | 1.327 | 0.184 | [-0.064, 0.331] |

*Note.* The standard deviation in random effects was 0.637.

# **Table S14**: Marginal coefficients and their standard errors obtained from the Beta GLMM fitted to the data of (block-level) scaled exit thresholds from both-instances of the patch-foraging task (*n* = 32).

| **Term** | **Estimate** | **Standard error** | **z** | ***p*-value** | **95% CI** |
| --- | --- | --- | --- | --- | --- |
| Intercept | 0.708 | 0.329 | 2.152 | 0.031 | [0.063, 1.354] |
| Travel time  ***[10-3]*** | -0.411 | 0.067 | -6.148 | 0.000 | [-0.542, -0.28] |
| Environment Order  ***[shortLong-longShort]*** | -0.104 | 0.220 | -0.474 | 0.635 | [-0.536, 0.327] |
| Scaled trait anxiety | -0.347 | 0.667 | -0.520 | 0.603 | [-1.654, 0.961] |
| PA_unalike_ | -0.179 | 0.164 | -1.091 | 0.275 | [-0.5, 0.142] |
| NA_unalike_ | -0.399 | 0.192 | -2.079 | 0.038 | [-0.776, -0.023] |
| PA_median_ | -0.062 | 0.078 | -0.785 | 0.433 | [-0.215, 0.092] |
| NA_median_ | 0.123 | 0.093 | 1.316 | 0.188 | [-0.06, 0.306] |

# **Table S15**: Reference grid used to obtain adjusted predictions from the Beta generalized mixed model fitted to data of the first-instance of the patch-foraging task (n = 60).

| NA_unalike_ | Travel time | Environment order | TA | PA_unalike_ | PA_median_ | NA_median_ |
| --- | --- | --- | --- | --- | --- | --- |
| 0.00 | 3 | shortLong | 0.445 | 0.507 | 0.983 | 0.667 |
| 0.40 | 3 | shortLong | 0.445 | 0.507 | 0.983 | 0.667 |
| 0.50 | 3 | shortLong | 0.445 | 0.507 | 0.983 | 0.667 |
| 0.67 | 3 | shortLong | 0.445 | 0.507 | 0.983 | 0.667 |
| 0.83 | 3 | shortLong | 0.445 | 0.507 | 0.983 | 0.667 |
| 1.00 | 3 | shortLong | 0.445 | 0.507 | 0.983 | 0.667 |
| 0.00 | 3 | longShort | 0.445 | 0.507 | 0.983 | 0.667 |
| 0.40 | 3 | longShort | 0.445 | 0.507 | 0.983 | 0.667 |
| 0.50 | 3 | longShort | 0.445 | 0.507 | 0.983 | 0.667 |
| 0.67 | 3 | longShort | 0.445 | 0.507 | 0.983 | 0.667 |
| 0.83 | 3 | longShort | 0.445 | 0.507 | 0.983 | 0.667 |
| 1.00 | 3 | longShort | 0.445 | 0.507 | 0.983 | 0.667 |
| 0.00 | 10 | shortLong | 0.445 | 0.507 | 0.983 | 0.667 |
| 0.40 | 10 | shortLong | 0.445 | 0.507 | 0.983 | 0.667 |
| 0.50 | 10 | shortLong | 0.445 | 0.507 | 0.983 | 0.667 |
| 0.67 | 10 | shortLong | 0.445 | 0.507 | 0.983 | 0.667 |
| 0.83 | 10 | shortLong | 0.445 | 0.507 | 0.983 | 0.667 |
| 1.00 | 10 | shortLong | 0.445 | 0.507 | 0.983 | 0.667 |
| 0.00 | 10 | longShort | 0.445 | 0.507 | 0.983 | 0.667 |
| 0.40 | 10 | longShort | 0.445 | 0.507 | 0.983 | 0.667 |
| 0.50 | 10 | longShort | 0.445 | 0.507 | 0.983 | 0.667 |
| 0.67 | 10 | longShort | 0.445 | 0.507 | 0.983 | 0.667 |
| 0.83 | 10 | longShort | 0.445 | 0.507 | 0.983 | 0.667 |
| 1.00 | 10 | longShort | 0.445 | 0.507 | 0.983 | 0.667 |

*Note.* For obtaining adjusted-predictions, we used different values of predictor variables in the fitted Beta GLMM shown by the first three columns (from left) while fixing other terms (regressors) at specific values. TA: Min-max scaled trait anxiety score.

# **Table S16**: Bayesian posterior estimates of regression weights associated with regressors of the drift diffusion model.

| **Variable** | **Mean** | **Std** | **2.5q** | **25q** | **50q** | **75q** | **97.5q** | **MC error** |
| --- | --- | --- | --- | --- | --- | --- | --- | --- |
| a | 1.436 | 0.057 | 1.334 | 1.397 | 1.434 | 1.471 | 1.560 | 0.002 |
| v_Travel-time_ | 0.019 | 0.007 | 0.004 | 0.014 | 0.019 | 0.024 | 0.033 | 0.000 |
| v_Berry-count_ | 1.560 | 0.028 | 1.503 | 1.540 | 1.560 | 1.579 | 1.614 | 0.001 |
| v_Env.-order_ | 0.155 | 0.084 | -0.012 | 0.100 | 0.156 | 0.212 | 0.313 | 0.007 |
| v_PA-median_ | -0.004 | 0.117 | -0.260 | -0.082 | 0.013 | 0.073 | 0.220 | 0.011 |
| v_NA-median_ | 0.129 | 0.113 | -0.086 | 0.054 | 0.127 | 0.205 | 0.346 | 0.010 |
| v_PA-unalike_ | 0.158 | 0.232 | -0.352 | 0.027 | 0.165 | 0.320 | 0.572 | 0.022 |
| v_NA-unalike_ | 0.545 | 0.223 | 0.118 | 0.406 | 0.540 | 0.675 | 1.078 | 0.020 |
| v_Trait-anxiety_ | -0.094 | 0.087 | -0.258 | -0.154 | -0.093 | -0.042 | 0.094 | 0.007 |

# **Table S17**: Results of likelihood ratio tests.

| **Models** | **No. of**  **params.** | **AIC** | **logLikelihood** | $\boldsymbol{\chi}^{\boldsymbol{2}}$**-**  **statistic** | **df** | **P(>**${\boldsymbol{\chi}^{\boldsymbol{2}}}_{\boldsymbol{df}}$**)** |
| --- | --- | --- | --- | --- | --- | --- |
| Need to be with others (“Interaction-intensity”), responses from ‘Exit_R/E_’ (n = 88) | | | | | | |
| Need ~ TA + SC + (1 \| ID) | 7 | 4791.738 | -2388.869 | - | - | - |
| Need ~ TA * SC + (1 \| ID) | 8 | 4792.632 | -2388.316 | 1.106 | 1 | 0.293 |
| Need ~ TA + SC + (1 + SC \| ID) | 9 | 4722.319 | -2352.159 | 73.419 | 2 | < 0.001 |
| Need to be with others (“Interaction-intensity”), responses from ‘Exit_R_’ (n = 80) | | | | | | |
| Need ~ TA + SC + (1 \| ID) | 7 | 4348.844 | -2167.422 | - | - | - |
| Need ~ TA * SC + (1 \| ID) | 8 | 4349.897 | -2166.948 | 0.947 | 1 | 0.33 |
| Need ~ TA + SC + (1 + SC \| ID) | 9 | 4285.278 | -2133.639 | 67.566 | 2 | < 0.001 |
| Type of interaction (“Interaction-with”), responses from ‘Exit_R/E_’ (n = 88) | | | | | | |
| IW ~ TA + SC + (1 \| ID) | 5 | 3369.926 | -1679.963 | - | - | - |
| IW ~ TA * SC + (1 \| ID) | 6 | 3371.845 | -1679.923 | 0.081 | 1 | 0.776 |
| IW ~ TA + SC + (1 + SC \| ID) | 7 | 3361.946 | -1673.973 | 11.981 | 2 | 0.003 |
| Type of interaction (“Interaction-with”), responses from ‘Exit_R_’ (n = 80) | | | | | | |
| IW ~ TA + SC + (1 \| ID) | 5 | 3098.486 | -1544.243 | - | - | - |
| IW ~ TA * SC + (1 \| ID) | 6 | 3100.233 | -1544.117 | 0.253 | 1 | 0.615 |
| IW ~ TA + SC + (1 + SC \| ID) | 7 | 3094.241 | -1540.121 | 8.245 | 2 | 0.016 |
| Positive affect, responses from ‘Exit_R/E_’ (n = 88) | | | | | | |
| PA ~ TA * SC * IW + (1 \| ID) | 16 | 4526.781 | -2247.390 | - | - | - |
| PA ~ TA * SC * IW + (1 + SC \| ID) | 18 | 4496.438 | -2230.219 | 34.342 | 2 | < 0.001 |
| Positive affect, responses from ‘Exit_R_’ (n = 80) | | | | | | |
| PA ~ TA * SC * IW + (1 \| ID) | 16 | 4150.458 | -2059.229 | NA | NA | NA |
| PA ~ TA * SC * IW + (1 + SC \| ID) | 18 | 4124.817 | -2044.408 | 29.641 | 2 | < 0.001 |
| Negative affect, responses from ‘Exit_R/E_’ (n = 88) | | | | | | |
| NA ~ TA * SC * IW + (1 \| ID) | 16 | 3681.719 | -1824.860 | - | - | - |
| NA ~ TA * SC * IW + (1 + SC \| ID) | 18 | 3655.690 | -1809.845 | 30.03 | 2 | < 0.001 |
| Negative affect, responses from ‘Exit_R_’ (n = 80) | | | | | | |
| NA ~ TA * SC * IW + (1 \| ID) | 6 | 3341.860 | -1654.930 | - | - | - |
| NA ~ TA * SC * IW + (1 + SC \| ID) | 8 | 3314.795 | -1639.397 | 31.065 | 2 | < 0.001 |
| Variability in positive affect, responses from ‘Exit_R/E_’ (n = 87) | | | | | | |
| PA_unalike_ ~ TA + SC + (1\| ID) | 7 | -27.369 | 20.684 | - | - | - |
| PA_unalike_ ~ TA * SC + (1\| ID) | 8 | -26.614 | 21.307 | 1.245 | 1 | 0.264 |
| Variability in positive affect, responses from ‘Exit_R_’ (n = 79) | | | | | | |
| PA_unalike_ ~ TA + SC + (1\| ID) | 7 | -28.248 | 21.124 | - | - | - |
| PA_unalike_ ~ TA * SC + (1\| ID) | 8 | -27.273 | 21.636 | 1.025 | 1 | 0.311 |
| Variability in negative affect, responses from ‘Exit_R/E_’ (n = 87) | | | | | | |
| NA_unalike_ ~ TA + SC + (1\| ID) | 7 | 87.834 | -36.917 | - | - | - |
| NA_unalike_ ~ TA * SC + (1\| ID) | 8 | 88.931 | -36.465 | 0.093 | 1 | 0.342 |
| Variability in negative affect, responses from ‘Exit_R_’ (n = 79) | | | | | | |
| NA_unalike_ ~ TA + SC + (1\| ID) | 7 | 84.634 | -35.317 | - | - | - |
| NA_unalike_ ~ TA * SC + (1\| ID) | 8 | 86.152 | -35.076 | 0.482 | 1 | 0.487 |

*Note.* When comparing goodness-of-fit of random-intercepts only models, we observed that models without the interaction of trait anxiety (TA) and social context (SC) had always better fit to data. Thus, we always compared model with random slopes for social context, wherever it converged, with random-intecepts only model containing additive terms for TA and SC.

# **Table S18**: Preregistration deviation disclosure

| **Deviations** | | | | | | |
| --- | --- | --- | --- | --- | --- | --- |
| # | Details | | | Original Wording | Deviation Description | Reader Impact |
| 1 | Type | Sample | | “We will exclude participants … 3) who test positive at the end of the mandated quarantine period, which will preclude them from the recovery period observations, ….” | We initially assumed that participants would complete the quarantine survey only after receiving their COVID-19 test results. However, we later observed that some participants completed the survey within a few hours of being tested and therefore could only report the date of testing, not the date of result receipt. This issue became apparent only after data collection was complete, during preprocessing when we attempted to partition the study timeline into quarantine and post-quarantine phases.  We chose not to exclude these participants solely on this basis, as we at least had reliable information about their test dates. Given that testing procedures were likely implemented in coordinated batches to ensure smooth campus operations and the timely return of new student cohorts, we assumed reasonable temporal consistency in testing schedules across participants.  Accordingly, participant exclusion was determined using the exit-day imputation procedure (Figure Sx). | The impact of this deviation on the interpretation of the study’s results is minimal. We report model estimates both with and without the exit-day imputation procedure, allowing readers to directly assess the robustness of our findings to this analytic choice. This provides transparency regarding the extent to which conclusions depend on the assumption that the imputation procedure accurately estimated quarantine exit dates for participants who reported them. |
|  | Reason | New knowledge | |  |  |  |
|  | Timing | After data access | |  |  |  |
| 2 | Type | Covariates | | “We will analyse…3) if social setting (during and after quarantine), trait anxiety, fluctuations in happiness/ anxiety/ stress levels affect patch leaving time in the foraging game.” | In the preregistration, we stated that we would examine whether fluctuations in happiness, anxiety, and stress predict patch-leaving time, but we did not explicitly specify how fluctuations would be operationalised. In the present study, we quantified affective variability using the coefficient of unalikeability, which is appropriate for ordinal data. This choice was motivated by the ordinal nature of EMA survey responses and by the observation that treating these responses as continuous in linear mixed-effects models violated model assumptions.  Alternative operationalisations of variability (e.g., standard deviation or mean-squared successive differences) are possible and may yield quantitatively different estimates. Thus, this represents a deviation from a fully specified preregistered analytic plan. However, the deviation concerns the operational definition of variability rather than the underlying hypothesis regarding affective fluctuations and patch-leaving behavior. | This deviation may influence the magnitude of reported effects insofar as different variability metrics capture distinct properties of affective dynamics. However, the central hypothesis—that affective fluctuations are associated with patch-leaving behavior—remains unchanged. The interpretation of results therefore depends on the appropriateness of treating EMA responses as ordinal and using an ordinal-consistent variability metric. |
|  | Reason | Typo/Error | |  |  |  |
|  | Timing | After data access | |  |  |  |
| 3 | Type | Hypotheses | | “3) Sleep quality will deteriorate during quarantine concomitant with increased negative affect and stress.” | Although we preregistered the hypothesis that sleep quality would deteriorate during quarantine alongside increased negative affect and stress, we did not perform this analysis. Testing this hypothesis would have required substantial additional analyses and reporting, thereby considerably broadening the scope of the paper beyond its primary focus on affective dynamics, social context, trait anxiety, and patch-foraging decisions. Because the sleep-related hypothesis is conceptually separable from the main decision-making framework examined here, we chose to reserve it for a dedicated analysis to ensure appropriate depth and clear presentation. We intend to pursue these sleep-related analyses separately and will report them in a dedicated manuscript. | The sleep-related hypothesis is analytically and conceptually separable from the primary decision-making analyses presented here. Therefore, its omission does not affect the interpretation of the reported associations among affect, social context, trait anxiety, and foraging behavior. The deviation limits the scope of inference to these domains and does not introduce bias into the reported results. |
|  | Reason | Other (Please Explain) | |  |  |  |
|  | Timing | After data access | |  |  |  |
| 4 | Type | Hypotheses | | “5) Female participants as compared to male participants will experience higher levels of negative affect both during and after the quarantine period.” | We did not test the gender-related hypothesis because female participants were substantially underrepresented in the raw EMA dataset (36 of 115 participants). This imbalance would have resulted in limited statistical power and unstable estimates for gender-based comparisons. We therefore refrained from conducting this analysis to avoid drawing potentially unreliable inferences. | Because gender was not included as a factor in the primary preregistered analyses, omitting the gender-related hypothesis does not affect the interpretation of the main findings. However, we acknowledge that this limits conclusions regarding potential gender differences and note this as a limitation. |
|  | Reason | Other (Please Explain) | |  |  |  |
|  | Timing | After data access | |  |  |  |
| **Unregistered Steps** | | | | | | |
| # | Details | | | Original Wording | Unregistered Step Description | Reader Impact |
| 1 | Type | | Analysis | “We will analyse…3) if social setting (during and after quarantine), trait anxiety, fluctuations in happiness/ anxiety/ stress levels affect patch leaving time in the foraging game.” | In the preregistration, we did not specify which EMA surveys preceding the task would be used to estimate affective fluctuations.  Prior to peer review, we operationalised pre-game affect using routine EMA responses collected within the 24-hour window before task onset, requiring at least two surveys to compute the coefficient of unalikeability.  Post peer review, we examined whether results were sensitive to the length of this pre-game window (e.g., 12h, 24h, 36h). We observed that window duration influenced the strength of associations between regressors and patch-foraging behavior metric. To provide a principled and behaviorally meaningful boundary, we defined the pre-game window as beginning at the participant’s wake-up time on the task day (Figure Sx), thereby restricting affect estimates to the waking period most proximal to task performance.  We report how model estimates vary across alternative window definitions to allow readers to assess robustness. | Because affective fluctuation estimates depend on the temporal window used for aggregation, different window definitions yield quantitative differences in effect size. However, by transparently reporting results across multiple window specifications, we enable readers to evaluate the robustness of the core association between affective variability and patch-leaving behavior. |
|  | Timing | | After results known |  |  |  |
| 2 | Type | | Hypotheses | - | We tested whether variability in affect was associated with social context and trait anxiety using ordered Beta generalized linear mixed models (GLMM). Variability was computed with coefficient of unalikeability using routine EMA survey responses. | Affect variability was one of the preregistered predictors in the patch-foraging analysis, but we did not hypothesize it to be associated with foraging behavior. After we observed that increased negative affect variability was associated with a lower exit threshold, we were intrigued to understand the associations among trait anxiety, social context, and affect variability.  We believed that assessing variability in affect beyond the pre-game window could guide us in future studies to hypothesize results with more precise directionality of effects between trait anxiety, social context, average affect responses, and their variability, and foraging behavior.  Most importantly, this exploratory exercise does not introduce bias into other EMA and foraging task analyses reported in the study. |
|  | Timing | | After data access |  |  |  |

# **Table S19**: Estimated fixed-effect coefficients of Ordered Beta GLMMs fitted to coefficients of unalikeability of affect variable (positive and negative affect)

| **Data &**  **variable**  **Term** | **Exit_R/E_**  PA_unalike_ | **Exit_R_**  PA_unalike_ | **Exit_R/E_**  NA_unalike_ | **Exit_R_**  NA_unalike_ |
| --- | --- | --- | --- | --- |
| TA | 0.027 (p=0.943)  [-0.733,0.788] | -0.004 (p=0.991)  [-0.774, 0.765] | 1.646 (p=0.000)  [0.867, 2.425] | 1.599 (p=0.000)  [0.789, 2.409] |
| SC [PQ] | -0.175 (p=0.060)  [-0.357, 0.007] | -0.148 (p=0.124)  [-0.337,0.041] | -0.030 (p=0.789)  [-0.249, 0.189] | -0.031 (p=0.791)  [-0.259,0.198] |
| Num. of obs. | 174 ( = 87 $\times$ 2) | 158 (= 79 $\times$ 2) | 174 (= 87 $\times$ 2) | 158  (= 79 $\times$ 2) |
| R^2^ Marg. | 0.027 | 0.020 | 0.246 | 0.235 |
| R^2^ Cond. | 0.756 | 0.756 | 0.619 | 0.639 |
| AIC | -27.4 | -28.2 | 87.8 | 84.6 |
| BIC | -5.3 | -6.8 | 109.9 | 106.1 |
| ICC | 0.7 | 0.8 | 0.5 | 0.5 |

*Note.* Each Ordered Beta GLMM had following specification: XA_unalike_ ~ TA + SC + (1| ID) where XA was either positive or negative affect. We excluded one participant from both Exit_R/E_ (n = 88) and Exit_R_ (n = 80) before fitting the models as the participant had answered only one survey during quarantine, which made coefficient of unalikeability undefined for the quarantine period. Values in square brackets denote 95% confidence interval aroud the estimated coefficient. Exit_R/E_: dataset generated using reported or estimated quarantine exit day, Exit_R_: dataset generated using only reported quarantine exit day, TA: min-max scaled trait anxiety score, SC: social context, PQ: post-quarantine.

# Section 1: Supplementary notes

A 24/7 technical support contact was made available to the participants to address any questions or issues during the study.

## **S1.1 Baseline survey.** Along with STAI (Form Y2), the baseline survey consisted of the 50-item International Personality Item Pool (IPIP) version of the Big Five markers (1), the General Self-Efficacy Scale (2), and the Perceived Stress Scale (3) along with additional questions assessing general sleep quality and feelings of loneliness.

## **S1.2 Quarantine exit.** As per institutional policy, students returning to campus were required to have received at least one dose of a COVID-19 vaccine taken at least one month prior to arrival on campus. Additionally, the institute administered COVID-19 testing of students following three days in quarantine.

To track participants’ testing and quarantine exit status, a Quarantine Survey was administered via the Ethica app (see Table S2 for survey schedule). This survey remained active until participants submitted a response. We asked participants to report the test date, test result and exit day from quarantine in the survey. However, due to uncertainties in the timing of COVID-19 testing and the notification of test results, some participants completed the Quarantine Survey before receiving their test outcome. As a result, for a subset of participants (21 out of 116), direct data on quarantine exit day was unavailable (see Table S3).

Since data quality was monitored only after the completion of the study, we retrospectively estimated the quarantine exit day for these participants based on their reported test day and the typical exit timing of other participants within the same batch. Details of this estimation procedure are provided in Table S4.

## **S1.3 Sleep survey.** The sleep survey could be completed in 1–2 minutes and included questions on sleep duration (“How long did you sleep?”), sleep quality (“How was your sleep last night?”), and disturbances or interruptions during sleep.

## **S1.4 Study compensation.** Participants, except for those in Batch B1, received ₹30 (₹ = Indian rupee) as compensation for completing the baseline survey to encourage compliance.

Participants could earn up to ₹740 based on their adherence to study procedures, including completing EMA surveys and playing the foraging task. Specifically, participants were eligible to receive ₹290, prorated according to their EMA survey response rate (₹290 × response rate), along with a completion bonus of ₹150 (for response rates between 70% and 90%) or ₹250 (for response rates above 90%).

Additionally, participants earned ₹70 for completing the foraging game once, according to the instructions for either the quarantine or post-quarantine setting.

# Section 2: EMA data

## **S2.1 Exclusion criteria for EMA data.** We initially collected data from 116 university students. Participants were excluded from the analyses if they: (1) completed less than 40% of the scheduled routine EMA surveys during the study period, or (2) had reported insufficient information because of which we could not estimate or confirm their quarantine-exit day (Figure 2). After applying these criteria, the final sample consisted of 88 participants, contributing approximately 21,000 momentary observations. This sample captured a broad range of the trait anxiety spectrum, with STAI-Y2 scores ranging from 29 to 72. Details about days spent in quarantine (reported or estimated) by the participants are shown in Table S5.

EMA compliance in the final sample was satisfactory (M = 77%, SD = 15%) and was not associated with trait anxiety (slope = 0.067 ± 0.156, p = 0.666).

## **S2.2 Statistical analysis of EMA data**

**Model selection.** To assess whether trait anxiety moderated the effects of social context on need to interact (“Interaction-intensity”) and type of social interactions (“Interaction-with”), we compared models (see Methods, equations (1) and (2)), fitted to the data from ‘Exit_R/E_’, using likelihood ratio tests. For both “Interaction-intensity” and “Interaction-with”, the interaction term of social context and trait anxiety was not statistically significant (“Interaction-intensity”: logLikelihood_eq.1_ = -2388.9, logLikelihood_eq.2_ = -2388.3, $\chi_{(1)}^{2}$= 0.293 ; “Interaction-with”: logLikelihood_eq.1_ = -1680.0, logLikelihood_eq.2_ = -1679.9, $\chi_{(1)}^{2}$= 0.776).

To assess the impact of quarantine exit imputation procedure on model estimates, we refitted all models to the response data generated using ‘Exit_R_’ (see Tables S6-S9 and Table S19; model term ‘ID’ in the tables is same as the term ‘Participant’ mentioned in the model formulae in Methods).

Comparisons of goodness-of-fit using likelihood ratio tests of all models fitted to the responses of “Interaction-intensity”, “Interaction-with”, positive and negative affect as well as their coefficients of unalikeability for both ‘Exit_R/E_’ and ‘Exit_R_’ are reported are Table S17.

# Section 3: Foraging task

## **Section 3.1 Paradigm.**

**Task overview**. The task consisted of two blocks; each block lasted for 4 mins. Participants harvested berries from a series of patches by making a harvest or leave decision on each trial. Patches were placed closer to each other to represent a shorter travel time of 3 seconds in the ‘short’ block as compared to the longer travel time of 10 seconds in the ‘long’ block. Participants were not informed explicitly about the travel times.

**Task timeline (see Figure 1c: a1 - a7).** Participants had to move the cursor to a fixation square (placed centrally on the middle path) and hold it there for 1 s before they could make a choice *(a1)*. After 1 s, the fixation square filled with green color (*a2*) and participants could move the cursor to a patch, placed horizontally on the same level as that of the fixation square, to get berries (*a3*) or decided to leave it for a new one (*a6*). If the participant moved the cursor away before the square turned fully green, the fixation would reset, requiring them to attempt again. Upon harvesting the patch, a reward *r* was shown as a number rounded to two decimal places after a delay of 500 ms right after the cursor contacted the patch (*a3*). After 1 s of such contact, the hollow fixation square reappeared on the screen (*a1* & *a4*). To leave the patch, participants had to choose a hollow square placed horizontally opposite the patch. Leaving the patch initiated an animation that resembled walking in the upward direction (*a7*). Depending on the block, participants then waited for either 3 seconds or 10 seconds to arrive at a new patch. The total number of berries collected in the block was displayed at the end of the block. Upon completing the task, participants filled out a brief online survey (administered via Google Forms) to report their understanding of the task and task-related subjective experiences.

Participants were instructed to play the foraging task twice: once during quarantine and once after exiting quarantine. The task was hosted on Amazon Web Services (AWS), and the web link of the task was emailed to participants no earlier than the second and ninth days of their study period. Participants were instructed to complete the task in full-screen mode on their personal computers. They were explicitly informed that their monetary bonus at the end of the study would be directly proportional to the number of berries collected during the task, thereby encouraging them to maximize their berry collection.

**Block order.** Participants were assigned different block orders based on their batch: Batches B1 and B2 played the short block first, followed by the long block (‘shortLong’ arrangement). Batches B3, B4, B5, B6, and B7 played the long block first, followed by the short block (‘longShort’ arrangement).

## **Section 3.2 Error correction in the measurement of the first trial duration.** Let TD_i, p_ be the trial duration of the *i*^th^ decision in the *p*^th^ patch from a given instance of the patch-foraging task. The code written for collecting task data as participants played the patch-foraging task did not correctly measure the trial duration of the first decision (TD_1, 1_) i.e., if a participant played a 'longShort' arrangement in the first game instance, the first trial duration in the 'long' block was incorrectly measured.

TD_1, p_ (for all *p*) was defined as time elapsed between reaching a new patch and moving cursor to a patch image to harvest (or the leave square) after fixating on the central square. TD_i, p_ (for *i* ≠ 1 for all *p*) was defined as the the time elapsed between two consecutive choices (stay-stay or stay-leave).

Given this difference between the first and non-first decisions, we used median of the differences between reaction times and trial duration of the first decision across all patches (except the first patch) from the first block played as an estimate for the true TD_1, 1_.

## **Section 3.3 Data exclusion for the first-game-instance of the patch-foraging task.**

For the analysis of patch-foraging decisions, we applied the following exclusion criteria sequentially:

1. **Device Type**: Participants were required to have played the foraging game at least once on a laptop or desktop computer (and not on a mobile device). After applying this criterion, 96 participants remained in the data.
2. **Minimum Decisions**: Considering each participant’s first instance of gameplay, we required that participants make at least 20 decisions in both the ‘short’ block and the ‘long’ block. This requirement reduced the sample to 88 participants.
3. **EMA Data Availability**: To allow calculation of the coefficient of unalikeability and median affect ratings, participants had to have responded to at least 2 routine EMA surveys within the 24-hour window preceding their first game instance. After this step, 76 participants remained in the data.
4. **Trial Duration Outliers**: We defined trial duration as either (i) the time taken to make a choice after reaching a new patch, or (ii) the time elapsed between two successive choices within a patch (see Section 3.2 above). Thus, the sum of trial durations in a patch corresponded to the total time spent in that patch.

   We first corrected measurement error in first trial durations (see Section 3.2 above). Participants were excluded if their mean trial duration in a block fell outside the range defined by the sample-level mean ± 2 standard deviations of trial durations, calculated using participant-level mean values for the corresponding block. Applying this criterion led to the exclusion of 10 additional participants (hereafter referred to as **outliers**), yielding a sample of 66 participants for the foraging analyses.

   The distributions of average trial durations for the sample (n = 66) and for the excluded outliers (n = 10) are presented in Figure S7. Figure S8 further illustrates the trials that contributed to extreme average trial durations leading to participant exclusion.

## **Section 3.4 Data transformation.** We removed six participants from the sample of 66 participants as we could not estimate at least one of the two block-level exit thresholds for them, yielding a sample of 60 participants (see Methods, Statistical analysis of the first-game-instance).

The patch-level exit threshold was theoretically bounded between 0 and 7.719 because of the stochastic reward function used in the task (see Figure S10). The boundedness of exit threshold warranted the use of mixed models which have support over a bounded set of non-negative real numbers and can handle correlations between within-participant thresholds. One such model is a Beta generalised linear mixed-effects model (GLMM) which requires the target variable to be bounded between 0 and 1 (both ends exclusive). To model the thresholds with a Beta GLMM, we first transformed the block-level thresholds, $u_{exit}$, using its theoretical maximum and minimum values:

$\begin{aligned} \hat{u}_{exit}=\frac{u_{exit}}{7.719}\#... \left( S1 \right) \end{aligned}$

Because one participant had harvested all patches in a block until they received multiple zero berries before leaving (see Figure 4b), and thus had $\hat{u}_{exit}= 0$, we further transformed $\hat{u}_{exit}$ such that it lies between $(0, 1)$ as suggested in (4):

$\begin{aligned} \bar{u}_{exit}=\frac{\hat{u}_{exit} *\left( N-1 \right)+0.5}{N}\#... \left( S2 \right) \end{aligned}$

where *N* (= 120) is the number of sample size.

## **Section 3.5 Model diagnostics.** The Beta GLMM (eq. (9) in Methods) was fitted to the data of 60 participants. We did not observe any convergence issues while fitting the specified model with R packages *glmmTMB* and *GLMMadaptive*. The estimates of the model fitted with *glmmTMB* were similar to that of with the model fitted with *GLMMadaptive*.

The estimated conditional modes of the random effects (and corresponding covariances) from the fitted model are reported in Figure S11. The estimated conditional modes of the random effects of four participants appeared to be extreme: all these participants had overharvested patches, on average, in the ‘long’ block. To understand why these participants had extreme block-level threshold, we visualised their data at patch-level (5). We observed that all these participants kept harvesting almost all patches (where they made more than two harvests) until they received almost zero berries. When we performed posterior predictive checks on the Beta GLMM, we observed that the model did not fit the data extremely well. We believed that this misfit rose because of the participants with extreme conditional modes of random effects (which was reflected as overharvesting in the ‘long’ block). Thus, we excluded these participants from the data and refitted the Beta GLMM while transforming $\hat{u}_{exit}$to $\bar{u}_{exit}$ (see eqs. S1 and S2) with N = 120 - 4 $\times$ 2 = 112 (see Methods, eq. (9)). The posterior predictive checks revealed a considerably good fit of the Beta GLMM to the data of 56 participants. The estimates of fixed-effect coefficients of this model are shown in Table S12.

## **Section 3.6 Reanalyses without quarantine exit uncertainty.**

## **A. Patch-foraging task.** To assess the impact of quarantine exit-day imputation (Supplementary Section 1.2), we reran the foraging analyses on the first-instance task data from only participants who explicitly reported their exit day (data ‘Exit_R_’). Using this restricted sample, we refitted the Beta GLMM to block-level exit thresholds. The pattern of results was qualitatively unchanged, with similar evidence for key effects, including the association of travel time and negative affect variability with exit thresholds (see Figure S13).

These findings indicate that the reported results are not driven by the exit-day imputation procedure for the data generated per the exclusion criteria of minimum 20 decisions in each block and completion of at least two routine EMA surveys in the pre-game window of 24 hr.

## **B. EMA outcomes.** We conducted analogous reanalyses for EMA outcomes (need to interact, type of interaction, positive and negative affect and their coefficients of unalikeability) using data from only participants who reported their quarantine exit day and had completed $\geq$ 40% of the routine EMA surveys i.e. data ‘Exit_R_’.

According to the likelihood ratio tests, random intercept models without the interaction term of trait anxiety and social context (eq. (1) in Methods) provided better fit to the data of ‘need to interact’ and ‘type of interaction’ compared to corresponding models containing the interaction term (eq. (2) in Methods), regardless of the use of exit-day imputation procedure (see Table S17). Thus, across these models, we observed that the strength of evidence for the associations between regressors and outcomes was similar to that reported in the main analyses (see Tables S6 & S7 for model estimates and Figure S14 for visual comparison). We made a similar observation in the case of CLMMs with random intercepts only (eq. (4) in Methods) fitted to positive and negative affect data (see Tables S8 and S9 for model estimates, and Figures S15 & S16 for visual comparison). This indicates that inference based on random-intercepts-only models is robust to the exclusion of imputed cases for EMA outcomes (Tables S6-S9).

## **Section 3.7 Sensitivity of foraging results to exclusion criteria and sample attrition.** To assess the impact of sequential exclusions on our findings (Table S12 & Figure 5c), we conducted a series of robustness analyses examining sensitivity to (i) influential participants (see Supplementary Section 3.5) and (ii) exclusion criteria (Supplementary Section 3.3):

**Transparency of exclusions**. A flow diagram summarizing all exclusion steps is provided in Figure 2, along with a comparison of included versus excluded participants on key characteristics (Figure S17), allowing direct evaluation of potential selection effects.

**Robustness to influential participants**. We refitted the Beta GLMM while retaining participants previously identified as having extreme decision-making strategies and computed robust standard errors. The resulting estimates and confidence intervals were similar to those obtained using standard model-based errors, with no changes in the signs of estimates or statistical significance of key effects (Figure S13). This indicates that the reported associations of block-level average exit threshold and regressors are not driven by a small number of influential observations.

**Sensitivity to exclusion criteria**. We next evaluated the sensitivity of the results to exclusion criteria by relaxing thresholds (minimum trial counts and EMA aggregation window) and re-estimating the Beta GLMM's model parameters across multiple datasets (Figure S17).

Across all specifications, the effect of travel time on exit thresholds remained consistently robust. In contrast, the association between variability in negative affect and exit thresholds was sensitive to analytic choices, reaching statistical significance in a subset of datasets (e.g., those generated using a 24-hour aggregation window), but not others (Figures S17 & S20).

Notably, when variability was computed using task-day EMA reports (see Figures S18 & S19), the association with exit thresholds was again observed, suggesting that this effect is strongest when affective measures are temporally proximal to task performance (Figures S17 & S20).

Together, these analyses indicate that while the primary task-related effect (travel time) is stable across different exclusion criteria, the relationship between variability in negative affect and foraging behavior is context-dependent and sensitive to data aggregation choices.

## **Section 3.8 Analysis of patch stay duration.** To complement analyses based on exit thresholds, we conducted confirmatory analyses using the preregistered outcome measure – stay duration (*psd*) in complete patches – with model formula as follows:

$$\begin{aligned} psd \sim TravelTime+EnvOrder+TA +{PA}_{median}+NA_{median}+ \\ PA_{unalike}+{NA}_{unalike}+\left( 1 | ID \right)\#... (3) \end{aligned}$$

Patch stay duration (*psd*) is strictly positive and right-skewed, violating the Gaussian assumptions of linear mixed-effects models. Consistent with this, linear mixed-effects models fitted to (raw and log-transformed) patch stay duration showed clear signs of model misspecification, including non-uniform DHARMa residuals (6, 7) and poor agreement between observed and simulated data (8) (Figures S21-S23). These results indicate that linear mixed-effects models are not appropriate to describe patch stay duration.

We therefore modeled patch stay duration using a Gamma generalized linear mixed model (GLMM) with a log link, which is well-suited for positive, skewed data. Model diagnostics indicated a good fit, with no systematic deviations in residuals and strong agreement between observed and simulated data (Figures S24 & S25).

Using the Gamma GLMM, we observed that travel time was positively associated with patch-stay duration, consistent with theoretical predictions from MVT and replicating the pattern observed using exit-threshold measures (Figures S26 & S27). Importantly, the association between variability in negative affect and stay duration was also observed, mirroring the pattern obtained in the primary analyses of block-level average exit thresholds (see Figure 5c & Figure S26).

Together, these results demonstrate that the key findings are consistent across both behavioral metrics, supporting the robustness of our conclusions and indicating that they are not dependent on the specific choice of outcome measure.

## **Section 3.9 Modeling of reaction time**

We fitted a Drift Diffusion Model (DDM) using the Python Toolbox HDDM (9) with the following formula:

$$\begin{aligned} v \sim1 + BerryCount + TravelTime+EnvOrder +{PA}_{median}+NA_{median}+ \\ PA_{unalike}+{NA}_{unalike}+TraitAnxietyScore\#... (4) \end{aligned}$$

The DDM was fitted to the trial-level data of complete patches from the same set of participants (n = 60) to which we had fitted the Beta GLMM (see Supplementary Section 3.4): these participants had harvested at least one patch with $\geq2$ harvests in each block. Trials with reaction times lesser than 0.1 s or greater than 5 s were removed (0.04% of 7396 trials).

## **Section 3.10 Analysis of both-game-instances of the patch-foraging task.**

There were 40 participants in the study who:

- played the patch-foraging task in quarantine as well as in non-quarantine setting on a laptop or a desktop computer (and not on a mobile phone),
- made at least 20 decisions in each of the four task blocks they played, and
- completed at least two rountine EMA surveys within the duration of 24 hours before each game-instance.

We then applied the following exclusion criteria sequentially:

1. **Trial Duration Outliers**: Participants were excluded if their average trial duration in a block fell outside the range defined by the mean ± 2 standard deviations, calculated using participant-level average trial durations for the corresponding block. Applying this criterion led to the exclusion of 6 participants, yielding a sample of 34 participants for the foraging analyses.
2. **Block-level reward exit threshold:** Estimating a block-level exit threshold is not possible for a participant if they did not harvest at least one patch twice in the block. There were two participants in the data set for whom we could not estimate exit thresholds for each of the four blocks.

The estimated block-level reward exit threhsolds ${(u}_{exit})$were then transformed to $\hat{u}_{exit}$ using eq. (1). Because no participant had harvested all patches in any block until they got multiple zero rewards, $\hat{u}_{exit}\in(0, 1)$. We fitted a Beta GLMM to the data of these 32 participants (see Methods, eq. (9) for model formula). The model estimates of Beta GLMM and marginal effects estimated from it are reported in Tables S13 and S14, respectively.

# Supplementary references

1.	L. R. Goldberg, The development of markers for the Big-Five factor structure. *Psychological assessment* **4**, 26 (1992).

2.	R. Schwarzer, M. Jerusalem, Generalized self-efficacy scale. *J. Weinman, S. Wright, & M. Johnston, Measures in health psychology: A user’s portfolio. Causal and control beliefs* **35**, 37 (1995).

3.	S. Cohen, G. M. Williamson, Perceived stress in a probability sample of the United States. (1988).

4.	M. Smithson, J. Verkuilen, A better lemon squeezer? Maximum-likelihood regression with beta-distributed dependent variables. *Psychological Methods* **11**, 54–71 (2006).

5.	G. A. Fox, S. Negrete-Yankelevich, V. J. Sosa, *Ecological statistics: contemporary theory and application* (Oxford university press, 2015).

6.	P. K. Dunn, G. K. Smyth, Randomized Quantile Residuals. *Journal of Computational and Graphical Statistics* **5**, 236–244 (1996).

7.	F. Hartig, DHARMa: Residual Diagnostics for Hierarchical (Multi-Level / Mixed) Regression Models. https://doi.org/10.32614/CRAN.package.DHARMa. Deposited 26 August 2016.

8.	A. Gelman, J. Hill, *Data Analysis Using Regression and Multilevel/Hierarchical Models*, 1st Ed. (Cambridge University Press, 2006).

9.	T. V. Wiecki, I. Sofer, M. J. Frank, HDDM: Hierarchical Bayesian estimation of the Drift-Diffusion Model in Python. *Front. Neuroinform.* **7** (2013).
